# Indol-3-ylglyoxylamide as Privileged Scaffold in Medicinal Chemistry

**DOI:** 10.3390/ph16070997

**Published:** 2023-07-12

**Authors:** Elisabetta Barresi, Marco Robello, Emma Baglini, Valeria Poggetti, Monica Viviano, Silvia Salerno, Federico Da Settimo, Sabrina Taliani

**Affiliations:** 1 Department of Pharmacy, University of Pisa, Via Bonanno 6, 56126 Pisa, Italy; elisabetta.barresi@unipi.it (E.B.); emma.baglini@farm.unipi.it (E.B.); valeria.poggetti@phd.unipi.it (V.P.); silvia.salerno@unipi.it (S.S.); federico.dasettimo@unipi.it (F.D.S.); 2 Laboratory of Bioorganic Chemistry, National Institute of Diabetes and Digestive and Kidney Diseases (NIDDK), National Institutes of Health, 9000 Rockville Pike, Bethesda, MD 20892, USA; marco.robello@nih.gov; 3 Department of Pharmacy, University of Salerno, 84084 Fisciano, Italy; mviviano@unisa.it

**Keywords:** indolylglyoxylamide, privileged structure, therapeutic effects, indole, glyoxylamide

## Abstract

In recent years, indolylglyoxylamide-based derivatives have received much attention due to their application in drug design and discovery, leading to the development of a wide array of compounds that have shown a variety of pharmacological activities. Combining the indole nucleus, already validated as a “privileged structure,” with the glyoxylamide function allowed for an excellent template to be obtained that is suitable to a great number of structural modifications aimed at permitting interaction with specific molecular targets and producing desirable therapeutic effects. The present review provides insight into how medicinal chemists have elegantly exploited the indolylglyoxylamide moiety to obtain potentially useful drugs, with a particular focus on compounds exhibiting activity in in vivo models or reaching clinical trials. All in all, this information provides exciting new perspectives on existing data that can be useful in further design of indolylglyoxylamide-based molecules with interesting pharmacological profiles. The aim of this report is to present an update of collection data dealing with the employment of this moiety in the rational design of compounds that are able to interact with a specific target, referring to the last 20 years.

## 1. Introduction

The concept of a “privileged structure” was proposed in 1988 by Evans and coworkers to define structural moieties able to furnish, with appropriate decorations, small molecules that are able to interact with different targets, receptors, or, more generally, proteins; actually, smart modifications of these structures could represent a fruitful strategy for the design and development of new receptor agonists/antagonists/allosteric modulators, enzyme inhibitors/activators, protein–protein interaction modulators, and so on [1].

**Figure 1:**
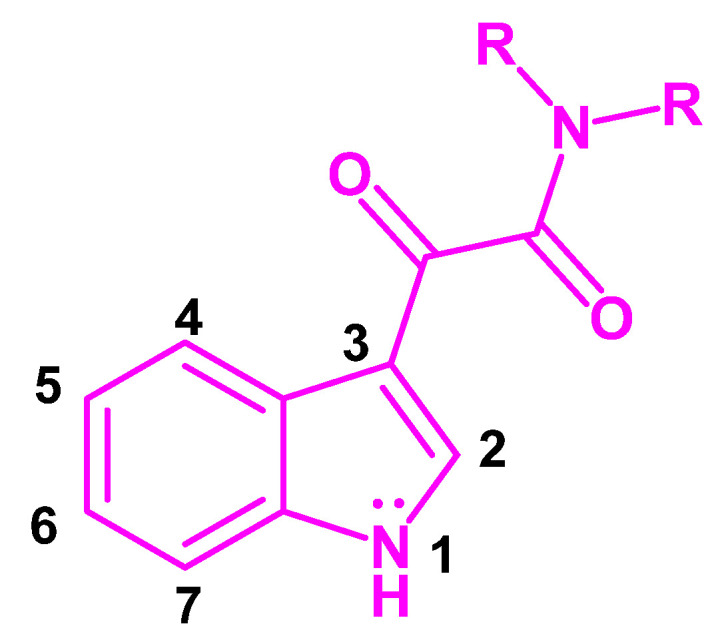
Structure of the basic indolylglyoxylamide scaffold.

The indole scaffold (Figure 1) emerges as one of the most important structural subunits for discovering novel drug candidates [2,3,4]. The evidence that indole constitutes a common structural feature of many biomolecules and natural products such as tryptophan, ergot alkaloids, and the neurotransmitter serotonin triggered extensive study of indole chemistry, leading to the development of a huge number of synthetic indole-based derivatives with biological activity. Many of them became drugs and others are useful for pharmacological purposes, but the majority became lead prototypes, validating the indole scaffold as a privileged structure [5].

A nitrogen lone pair is implicated in maintaining the aromaticity of the indole ring, with the nitrogen proton being very slightly acidic (pKa ~ 17) and able to engage in H-bond interactions with specific target proteins [5]. This interaction may be crucial to the anchoring of indole derivatives to certain molecular targets. As an example, Da Settimo and coworkers highlighted the crucial role of the indole N-H in a series of indolylglyoxylamides with activity on the central benzodiazepine receptor (BzR) [6]. Despite the literature indicating that the N-H group as a donor of hydrogen bonds was not required to have BzR affinity, it was shown that all derivatives featuring a benzofuran and benzothiophene nucleus possessed a lower affinity for the receptor compared to those bearing an indole nucleus [6].

In addition, the indole ring is able to establish noncovalent interactions due to its aromaticity, such as π–π stacking or cation–π interactions [7]. In this respect, two example studies of G protein-coupled receptors (GPCRs) were reported by Bondensgaard and coworkers [8] and Rad and colleagues [9], not only showing the crucial role of the indole scaffold in the molecular recognition of the target receptor but also, most importantly, proposing the biological validation of the indole substructure as a truly privileged scaffold for GPCR targets [8,9]. The concept of “biological validation” was used to distinguish “chemically privileged” substructures from privileged scaffolds that have overcome biological tests, gaining clinical significance [9].

The propensity of amides to establish hydrogen bonds has been extensively studied and exploited in crystal engineering [10]. In this respect, glyoxylamides provide a greater number of features with respect to a simple amide function. They introduce a greater degree of versatility to the system due to the possibility to form an additional hydrogen bond through the keto group and the variable glyoxylamide torsional angle [11].

The combination of the indole nucleus with the glyoxylamide function originated an excellent template, the indolylglyoxylamide moiety (Figure 1), which is suitable to a great number of structural modifications either to produce a specific therapeutic effect or to improve the pharmacokinetic (PK) profiles of biologically active molecules [12]. Actually, the synthetic ease and versatility of the indole nucleus and the potency attributed to glyoxylamides have led to the development of a wide array of indolylglyoxylamide compounds, which have shown different pharmacological effects in medicinal chemistry [12].

The purpose of this report is to provide an update of collection data dealing with the employment of indolylglyoxylamide moiety in the rational design of compounds able to interact with specific targets. Altogether, this information provides exciting new perspectives on existing data that can be useful in further design of indolylglyoxylamide-based molecules with interesting pharmacological profiles.

**Figure 2:**
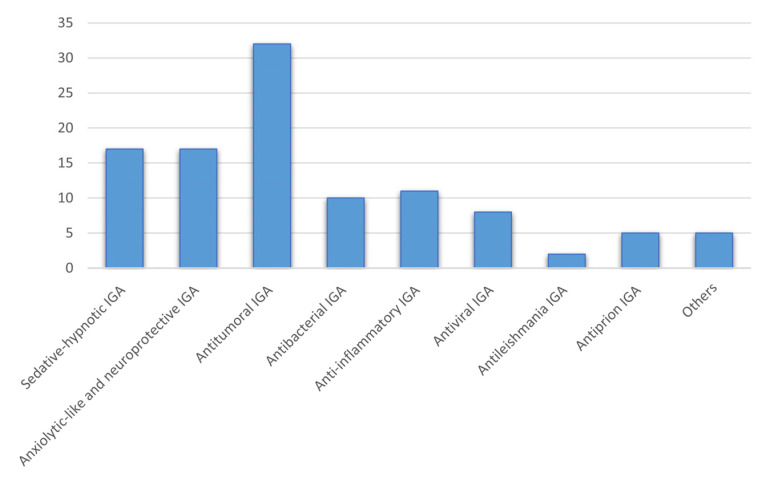
Graph representing the application of indolylglyoxylamides with different biological activity.

A synopsis of some of the applications of the indolylglyoxylamides in drug design encompassing different fields of biological activity is outlined in Figure 2.

## 2. Indolylglyoxylamides with Sedative–Hypnotic Properties

**Figure 3:**
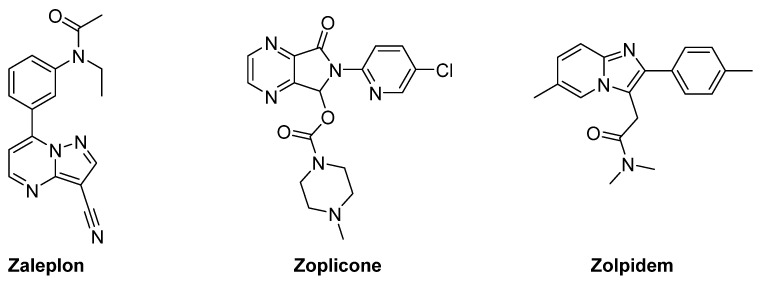
Structures of zaleplon, zoplicone, and zolpidem.

About 10% of the adult population suffers from insomnia, which is often a chronic condition. Insomnia increases with age, and women are more affected with respect to men. Only a few people seek medical help to treat insomnia, remaining unaware of behavioral and medical options available [13]. Barbiturates, often used as sedative hypnotics/anxiolytics, were the first generation of drugs to treat insomnia [14]. Benzodiazepines (Bzs), the second generation, have been used for the treatment of insomnia, and have, unlike barbiturates, lower potential for abuse and less danger of lethal overdose [15]. However, Bzs cause many adverse effects, including cognitive and psychomotor impairment, dependence, tolerance, hangover, and rebound insomnia [16]. Thus, a third generation of hypnotics, such as zolpidem, zopiclone, and zaleplon (Figure 3), was developed to obtain compounds endowed with sleep-inducing action combined with minor adverse effects, including amnesia and motor dysfunction [17]. During long-term treatment with zaleplon, eszopiclone, and a modified release formulation of zolpidem, neither tolerance during treatment nor limited rebound insomnia after therapy discontinuation was observed [18,19]. Unfortunately, when administered at higher doses, the same Bzs’ side effects were observed, whose severity depends on the specific drugs [20].

Bzs allosterically modulate the γ-aminobutyric acid (GABA) affinity for the type A GABA receptor (GABA_A_R), acting as agonists (positive allosteric modulators) endowed with anxiolytic, anticonvulsant, sedative-hypnotic, and myorelaxant effects.

The main Bz-sensitive GABA_A_R subtypes in the brain are α_1_β_3_γ_2_, α_2_β_3_γ_2_, α_3_β_3_γ_2_, and α_5_β_3_γ_2_, at variance with the α_4_β_3_γ_2_ and α_6_β_3_γ_2_ subtypes, which do not respond to Bzs [21,22,23,24]. The α subunit is the only one that influences the affinity and efficacy of BzR ligands, because the γ_2_ and β_3_ subunits do not vary [21,22,23,24].

Since 1980, numerous research groups have aimed at the development of new ligands with high affinity and selectivity for the different GABA_A_/BzR subtypes. In particular, selective α_1_ agonists should represented sedative–hypnotic agents without effects on learning or memory processes, selective α_2_/α_3_ agonists should possess anxiolytic activity without sedation, and selective α_5_ inverse agonists should constitute cognitive enhancers lacking anxiogenic activity [24].

Structure–affinity relationships (SARs) of structurally different classes of BzR ligands were rationalized by Cooks’ research group by means of a pharmacophore/topological receptor model constituted by (i) hydrogen-bond acceptor (A2), (ii) hydrogen-bond donors (H_1_ and H_2_), (iii) four lipophilic pockets (L1, L2, L3, and LDi), and (iv) three sterically forbidden sites (S1, S2, and S3) [25].

**Figure 4:**
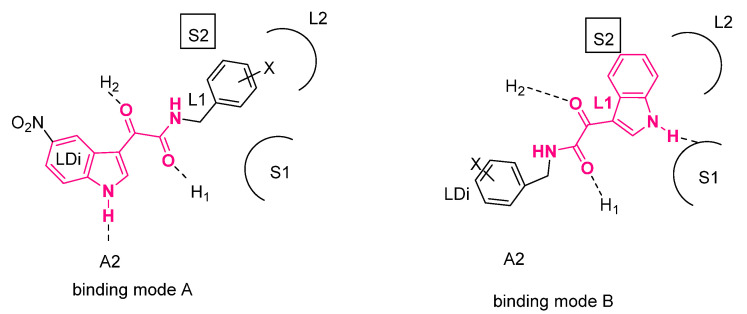
Hypothetical binding modes A and B of indolylglyoxylamide BzR ligands [27] within the framework of Cook’s pharmacophore/topological model [25]. A2 = hydrogen-bond acceptor; H1 and H2 = hydrogen-bond donors; L1, L2, L3, and LDi = lipophilic clefts; S1 and S2 = sterically forbidden sites; X = OH, OCH_3_, Cl.

Some years ago, Da Settimo and coworkers described a class of *N*-(benzyl)indol-3-ylglyoxylamide BzR ligands [26,27]. These compounds showed higher BzR α1-subtype affinity with respect to the α_2_ and α_5_ isoforms. SARs of this class indicated an interdependent effect of substituents at the 5-position and groups on the benzyl ring on α1 affinity. Affinity was favored when the benzyl ring was substituted with OH/OCH_3_ or halogens, depending on the substitution at the 5-position of the indole, in particular with a chlorine atom or with a nitro group, or not. Thus, whereas in the 5-unsubstitued series the most active compounds were those featuring an electron-attracting substituent (Cl), in the 5-Cl/NO_2_ series the activity was optimized with 3′,4′-(OH)_2_ or 3′,4′-(OCH_3_)_2_. These results evidenced the existence of two different binding modes, called A and B, for the interaction of these compounds within the receptor site (Figure 4) [27].

**Figure 5:**
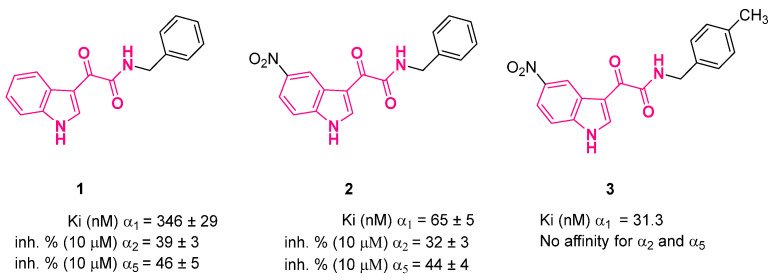
Indolyl-3-glyoxylamides **1**–**3** as sedative–hypnotic agents.

In a subsequent study, the authors exploited the difference in dimension between L2 and LDi lipophilic pockets to identify indolylglyoxylamides that were selective toward the different BzR subtypes [28]. Because the LDi and L2 clefts are wider in the α_1_ and α_5_ sites, respectively, compared to the other subtypes, full occupation of LDi or L2 may lead to α_1_ and α_5_ selective compounds, respectively [28,29]. Based on benzylindolylglyoxylamides **1** and **2** (Figure 5) as lead compounds [26], derivatives featuring different substituents on the benzylamide phenyl ring and compounds in which the benzyl moiety was replaced with alkyl groups were studied with the aim of probing the LDi and L2 pockets [28].

SARs evidenced how compounds featuring the same amide side chain showed different affinity depending on the substitution at the 5-position of the indole ring (NO_2_, H), confirming the ability of such compounds to adopt two binding poses (A and B) into the receptor binding site (Figure 5). The LDi cleft was wider than the L2 one; indeed, the former could host bulky 4′-substituents of 5-H indoles (binding mode B), whereas the latter could not accommodate the same 4′-substituents of the 5-NO_2_ counterparts (binding mode A). Some selected compounds were tested for their affinity at the α_1_, α_2_, and α_5_ BzR subtypes together with leads **1** and **2**, and all derivatives showed fair affinity and selectivity for α_1_ with respect to the α_2_ and α_5_ subtypes. Compound **3** showed the best α_1_ affinity and selectivity, displaying Ki = 31.3 nM at the α_1_ isoform and no affinity at the α_2_ or α_5_ isoforms [28]. In line with Cook’s BzR pharmacophore model, the data presented by Da Settimo and coworkers indicated how the full occupancy of the LDi region by a bulky lipophilic group shifted the selectivity and affinity of the ligand towards the α_1_ subtype, making the interactions with the L1/L2 lipophilic pockets contribute less to the binding and selectivity to other receptor subtypes. A functional assay performed on **3** evidenced a similar efficacy with respect to the standard diazepam in co-application with GABA at the BzR α_1_ subtype (72 ± 2% and 78 ± 3% for **3** and diazepam, respectively), showing that this compound acts as a full agonist at the α_1_ subtype [28]. Finally, in vivo behavioral assays based on the observation of the spontaneous motor activity of mice identified **3** as a sedative–hypnotic agent, although less active with respect to zolpidem [28].

## 3. Indolylglyoxylamides with Anxiolytic-Like and Neuroprotective Properties

As described above, Bzs are the first-line treatment for anxiety disorders, eliciting their action by allosterically binding the GABA_A_R. If their binding causes an enhancement in GABA inhibitory action, they are called agonists, whereas when it causes a GABA action reduction, they are called partial agonists. Finally, if their binding does not produce any effects, they are called antagonists [30]. Bzs are also classified as full agonists (or full inverse agonists) and as partial agonists (or full inverse agonists), depending on their efficacy toward the different GABA_A_R subtypes.

As Bzs cause several unwanted side effects, research has focused on the identification of safer anxiolytic agents. Due to their differential localization in brain areas, the different GABA_A_R subtypes may be associated with distinct physiological effects, specifically (i) α_1_ subtype moderating sedation, (ii) α_2_/(α_3_) subtype mediating anxiolytic and myorelaxation actions, and (iii) α_5_ subtype being involved with cognition processes [24,31,32].

Based on this, to develop an anxioselective compound [33], either selective binding agonism to α_2_ (affinity-based selective agents) or agonism to the α_2_ subtype and antagonism to other isoforms (efficacy-based selective agents) is required. In addition, anxioselective compounds lacking sedation and dependence may interact with all four GABA_A_R subtypes as partial agonists [30].

**Figure 6:**
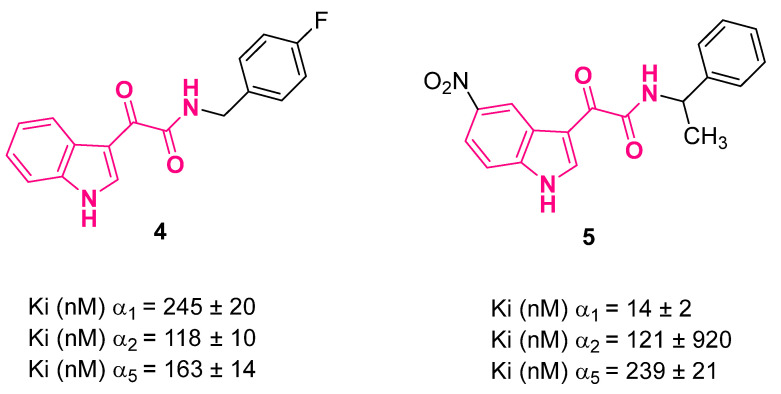
Indol-3-ylglyoxylamides **4** and **5** as anxioselective agents.

With respect to nonselective Bzs, either affinity- or efficacy-based α_2_ selective agonists should conserve anxiolytic properties with minor collateral effects, namely, sedation, ataxia, tolerance, dependence, and impairment of cognitive processes [24,31]. Aiming at developing novel anxioselective agents, Da Settimo et al. [27,28,34,35] studied a huge number of indol-3-ylglyoxylamides, and the major result was the identification of compounds **4** and **5** (Figure 6), which showed the highest potency at the α_2_-BzR together with an interesting in vitro profile (full α_2_ agonism, α_1_ partial agonism/antagonism, low affinity to α_5_). Such compounds were also assayed in vivo for their effects on mouse anxiety by means of a light/dark (LD) apparatus. The LD test is based on the mouse being conflicted between its natural hostility to brightly lit areas and its spontaneous tendency to explore novel environments. The most reliable parameters to assess anxiolytic-like activity have been reported to be the time spent in the illuminated area and the number of transitions between the light and dark areas [36]. Compounds **4** and **5** administrated in mouse i.p. at a dose of 20 mg/kg each determined an increase in the time spent in the white compartment (TW) and the number of transfers from the illuminated to the dark area and vice versa (Tran) values, thus representing potential anxiolytic agents without sedative activity.

**Figure 7:**
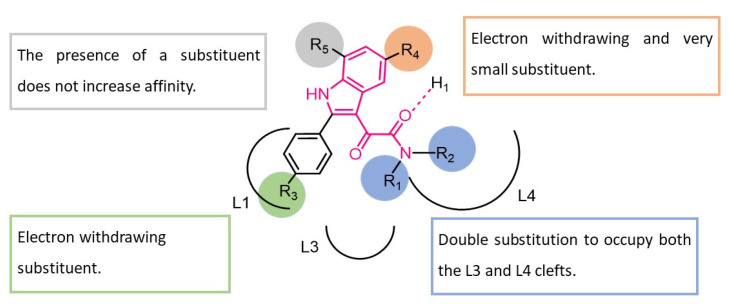
SAR of *N*,*N*-dialkyl-2-phenylindol-3-ylglyoxylamides (PIGAs) in the pharmacophore/topological model.

Recently, research has focused on ligands acting at different sites of the GABA_A_R in order to develop novel anxiolytic drugs endowed with improved safety profiles. Neurosteroids elicit anxiolytic effects favorable for memory, learning, and emotional processes without sedation by positively modulating GABA neurotransmission [37]. Of note, at the central nervous system (CNS) level, their concentration was enhanced by ligands of translocator protein (TSPO) [38], formerly known as the “peripheral benzodiazepine receptor,” such as diazepam, Ro5-4864, alpidem [39], PK11195 [40], and FGIN-1-27 [41,42]. TSPO ligands were shown to promote cholesterol translocation into mitochondria, where it is converted into pregnenolone, a steroid precursor [43]. In this connection, neurosteroidogenic TSPO ligands may be exploited to identify novel anxiolytic agents, avoiding the side effects typical of Bzs [39,44,45,46]. In light of the above, Taliani and coworkers [47,48,49,50] developed a class of *N*,*N*-dialkyl-2-phenylindol-3-ylglyoxylamides (PIGAs) potentially able to bind TSPO (Figure 7). Notably, these compounds were developed because of the structural similarity between indolylglyoxylamides, previously reported as BzR ligands with a sedative/hypnotic or anxiolytic/nonsedative profile [28,34,35], and 2-arylindol-3-acetamides, reported by Kozikowski et al. [42] to be TSPO selective high-affinity ligands. Numerous PIGAs showed subnanomolar affinity for TSPO together with the capacity to enhance pregnenolone concentrations in rat C6 glioma cells and in a human astroglioma cell line (U87MG) [47,48,49,51]. SARs of this class of compounds were rationalized by means of a pharmacophore/topological model composed of three lipophilic clefts (L1, L3, and L4) and an H-bond donor moiety (H_1_) (Figure 7). In detail, the subsequent interactions were supposed to be established: (i) an H-bond between the amide carbonyl and the H_1_ site, (ii) lipophilic interactions between the two hydrophobic groups R_1_ and R_2_ with the L3 and/or L4 clefts, and (iii) π-stacking interaction between the 2-phenyl group and the L1 pocket (Figure 7). Biological evaluations of a large number of PIGAs allowed the authors to delineate the following structural requirements for an optimal interaction with TSPO: (i) a double substitution on the amide nitrogen, (ii) an electron-withdrawing substituent at R_3_, and (iii) an electron withdrawing and very small substituent at R_4_. Finally, the presence of a substituent at the 7-position (R_5_) did not increase affinity (Figure 7).

In addition, a number of these compounds exhibited in vivo anxiolytic/nonsedative properties in rodents, which was related to their ability to stimulate neurosteroid production, which, in turn, caused a positive allosteric modulation of GABA_A_R [48,52].

**Figure 8:**
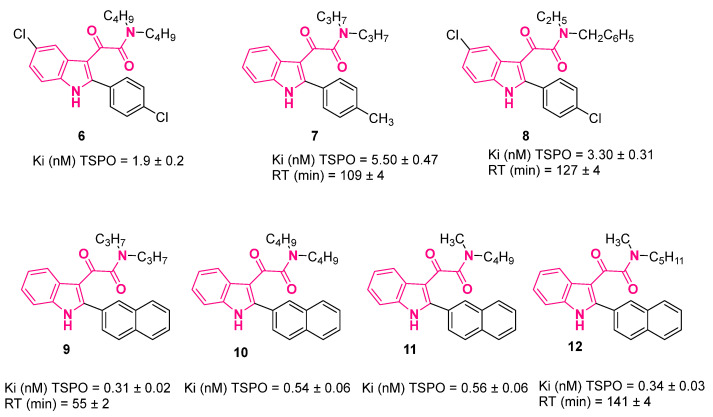
*N*,*N*-dialkyl-2-phenylindol-3-ylglyoxylamides (PIGAs) **6-12** as anxiolytic and neuroprotective agents.

A peculiar characteristic of several classes of TSPO ligands is the lack of correlation between the in vitro steroidogenic activity and binding affinity [53,54,55]. To resolve the gap in that connection, the residence time (RT) has been identified as the best parameter to correlate these two issues. Accordingly, the RT of some selected PIGAs, i.e., **6**–**12** (Figure 8), was quantified and the results highlighted a positive correlation between the steroidogenic efficacy and RT, unlike what observed between efficacy and affinity [51,56]. It was also observed that the main requirements to obtain high RTs and thus high-efficacy compounds were a highly lipophilic moiety at the 2-position of the indole together with at least one of the two *N*-alkyl groups on the amide nitrogen with a number of carbon atoms in the 1–3 range. In fact, PIGAs with in vivo anxiolytic-like properties in rats, namely, **7**, **8**, and **12** (Figure 8) [48,56], displayed long RT values, evidencing the important role played by RT in predicting the in vivo efficacy of TSPO ligands. To determine the structural reasons behind this difference in RT values, an enhanced sampling molecular dynamics simulation of compounds **6** and **12** (Figure 8), selected on the basis of their different kinetic parameters and efficacy in steroidogenesis, was carried out. The results clearly evidenced that slight structural differences of PIGAs had a significant impact on the unbinding energetics and thus on RT [57].

Very recently, compound **12** (Figure 8) was used to evaluate the immunomodulatory function of TSPO in human microglia [58]. Specifically, **12**-pretreatment of interleukin-1β-activated human C20 microglial cells, an adult-derived immortalized cell line that shows the typical phenotypic and functional characteristics of physiological microglia, induced a phenotypic shift from M1 and M2 by reducing the release of pro-inflammatory (IL-8) cytokines and increasing the release of anti-inflammatory (IL-4) ones [58].

Compounds **8** and **12** (Figure 8) were also evaluated for their effect on inflammatory-based retinal neurodegeneration in an in vitro model of lipopolysaccharide (LPS)-induced degeneration in 661W cells, a photoreceptor-like cell line [59]. Both compounds modulated the inflammatory and apoptotic processes in 661W cells and reduce LPS-driven cytotoxicity, effects that were shown to be mediated by neurosteroids [59].

Compound **12** (Figure 8) was shown to be also able to reduce myelin oligodendrocyte glycoprotein experimental autoimmune encephalomyelitis (MOG-EAE) disease progression, severity, and neuropathological markers by (i) promoting neurosteroid allopregnenolone synthesis in the CNS and (ii) increasing the production of anti-inflammatory IL-10, evidencing its potential use as a tool against primary progressive multiple sclerosis (PPMS) or severe multiple sclerosis (MS) [60].

Finally, compound **12** (Figure 8) was also employed to investigate the impact of neurosteroidogenesis on cholesterol homeostasis in an in vitro human microglia model, with cholesterol accumulation usually being associated with the impairment of neuroinflammatory response, favoring neurodegenerative disease progression. The results evidenced the crucial role played by TSPO ligands, such as **12**, in promoting neurosteroidogenesis, allowing for the restoration of cholesterol homeostasis and thus the maintenance of the correct functionality of microglia for the treatment of brain diseases related to neuroinflammation [61].

The biological activities of **12** are summarized in Table 1.

## 4. Indolylglyoxylamides with Antitumoral Properties

Cancer is the main cause of death worldwide, accounting for more than 19.3 million newly diagnosed cases. The widely recognized multistep process of carcinogenesis assumes that the accumulation of several mutations in an individual cell leads to significant modifications in its proliferation and differentiation behavior, reaching a malignant state with metastasis via benign intermediate stages [62]. Cancer therapy differs depending on the type and stage of the tumor, but it can be identified with common strategies, including surgery, which is the most suitable in the case of solid tumors, although it may favor the spreading of metastases [63]. Moreover, radiation (ionizing [64], thermal [65], and photodynamic [66]) can be used to treat tumors through what is known as radiotherapy, which, like surgery, is preferable for localized or isolated cancers [67]. Chemotherapy and immunotherapy are other possible ways of treating oncological diseases represented mainly by disseminated or systemic tumors [65]. The first is based on the use of “antineoplastic” substances, namely, chemical agents against new growth, whose cytotoxic action interferes with cellular synthesis, DNA and RNA functions, and life-sustaining proteins. The second one, discovered more recently, is among the most widely studied approaches due to its greater selectivity and effectiveness through immune stimulation by using monoclonal antibodies [68].

Despite massive research efforts, it has still not been possible to develop drugs able to trigger a marked prolongation of survival time or even a complete cure for widespread solid tumors. The main problems associated with cancer chemotherapy are the lack of, or poor response, to treatments currently available, severe side effects, and the onset in many tumors of a multi-drug resistance process, which can be considered the most severe complication of these diseases. Tumors that have developed resistance to more than one drug are said to be “multi-drug resistant” (MDR), and in this case there is little that can be done to halt or retard further progression of the disease.

Therefore, there is still a need to develop new drugs to overcome the aforementioned problems with the currently used treatments. Ideal anticancer drugs should be efficient against currently untreatable or poorly treatable tumors and against MDR tumors, with oral bioavailability and/or reduced side effects [69]. 

**Figure 9:**
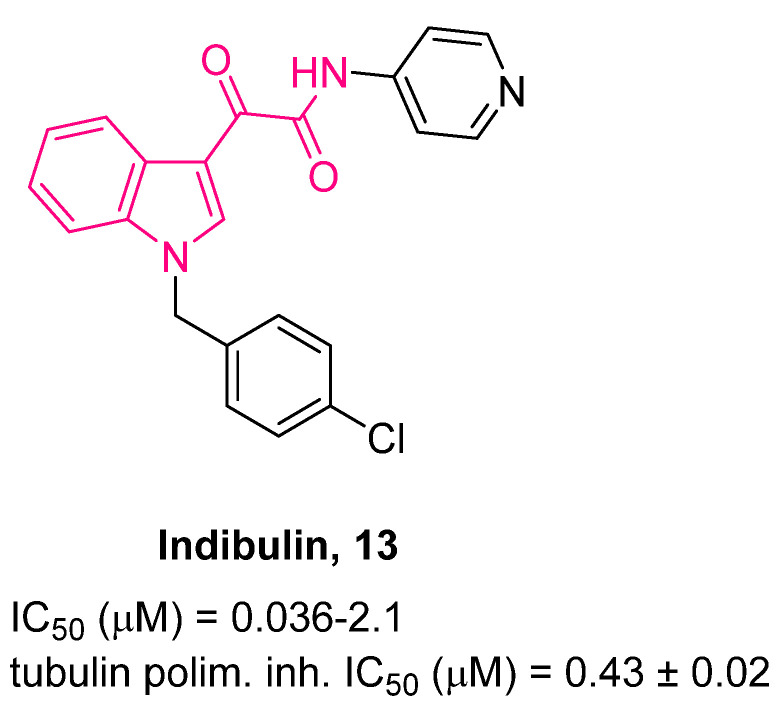
Structure of indibulin **13**.

Among the indol-3-ylglyoxylamides, indibulin **13** (ZIO-301; D-24851, Figure 9) is an orally applied, synthetic, small molecule whose antitumor effect is based on microtubule destabilization [70].

Indibulin **13** provokes cell accumulation with condensed nuclei and abnormal mitotic spindles and cell cycle arrest at metaphase; it exhibits anticancer activity against a broad spectrum of human tumor cell lines and xenografts, including MDR tumor cells and taxane refractory tumors [71]. In preclinical studies, **13** did not show neurotoxicity—which is usually observed with other tubulin binding drugs, i.e., taxanes and vinca alkaloids—because of the lack of affinity for microtubules in differentiated neuronal cells with post-translationally modified tubulin [72]. Furthermore, **13** exhibits good bioavailability after oral administration and therapeutic treatment at nontoxic doses [71]. Therefore, this antimitotic drug exhibits a notable improved therapeutic index with respect to other tubulin binding agents, i.e., paclitaxel and vincristine [73]. However, further clinical tests in a phase I trial [74] of an oral drinking solution of **13** in 10% lactic acid evidenced an increase in the occurrence of nausea and vomiting [74]. In 2010, Oostendorp et al. tested a capsule formulation of **13** to improve its tolerability [75]. The protocol was modified to twice daily dosing with the aim of further increasing the systemic exposure upon oral administration of **13**. Unfortunately, the results were not the expected ones, and the formulation still needs optimization.

**Figure 10:**
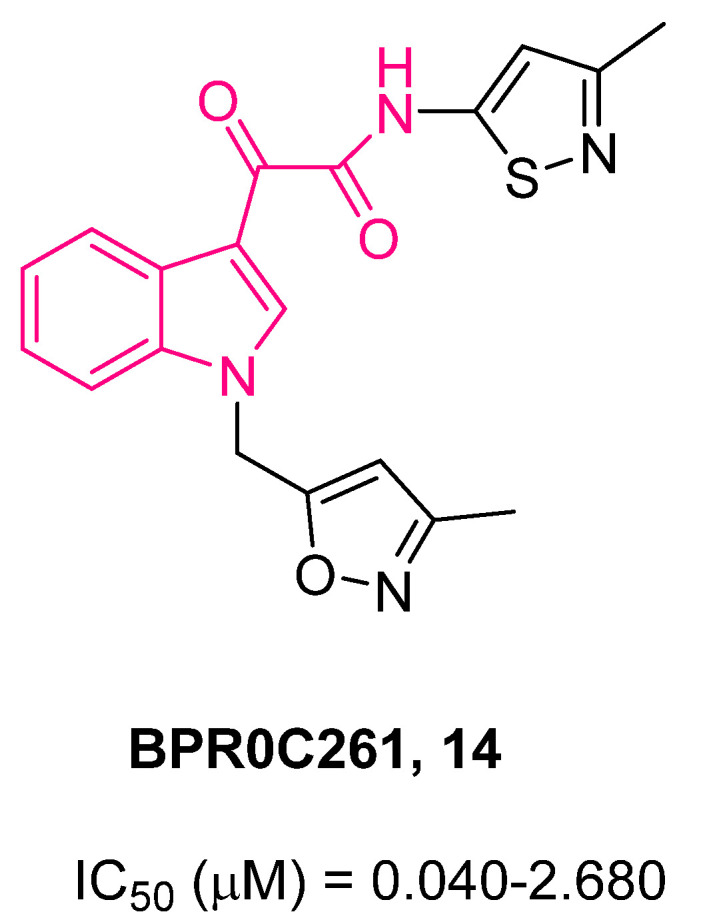
Structure of BPR0C261 **14**.

**Figure 11:**
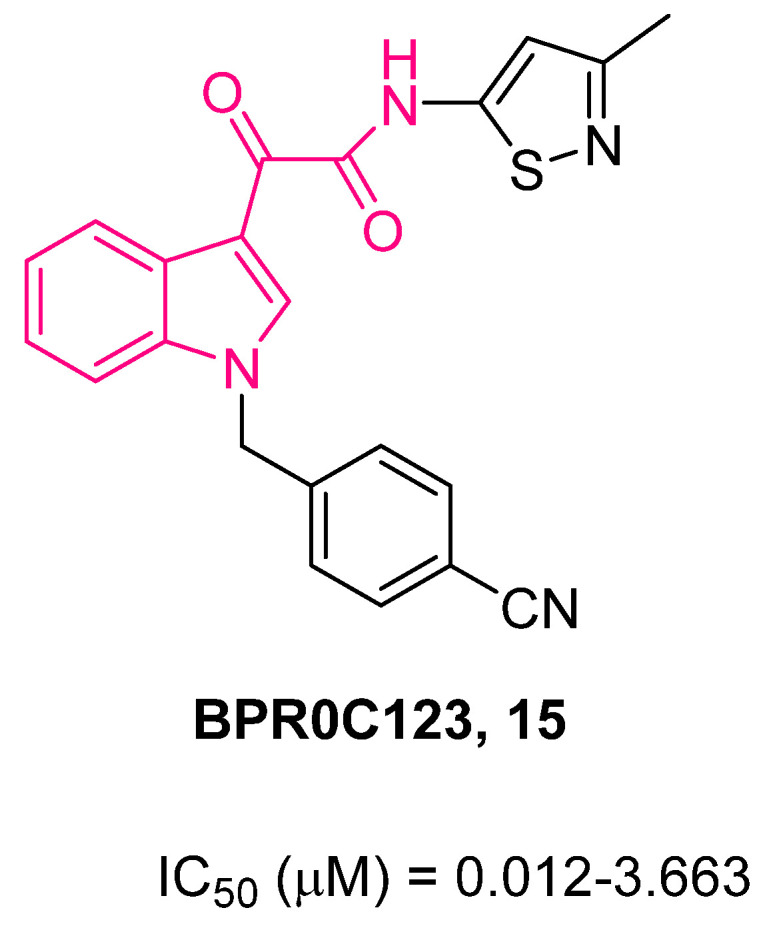
Structure of BPR0C123 **15**.

With the aim of investigating whether the replacement of the aryl rings with different heterocycles at the 1- and/or 3-positions of **13** may increase the biological activity and confer potential advantages in terms of pharmacological and PK features [76,77], Li et al. developed a series of *N*-heterocyclic indolylglyoxylamides [78]. In general, the replacement of the *p*-chlorophenyl and pyridine rings of **13** with different heterocyclic moieties increases the solubility of the molecules in water. The anticancer effect of such derivatives was assayed in vitro against six human cancer cell lines (gastric NUGC3, murine leukemic P388, hepatocellular HepG2, breast MCF7 and its doxorubicin (adriamycin)-resistant MCF7/ADR, uterus MES-SA and its doxorubicin-resistant MES-SA/Dx5 subline) and the results clearly demonstrated an enhanced anticancer activity of these compounds with respect to **13** [78]. A crucial role played by steric and electronic factors of the heterocycle at the 1-position was evidenced, whereas for the 3-position, the presence of an isothiazolyl ring appeared to be an important feature. The most active compounds were **14** and **15** (Figure 10 and Figure 11). Both compounds exhibited a similar dose-dependent in vivo activity at oral doses up to 100 mg/kg when tested on a leukemic P388 cancer survival model in young female inbred DBA/2J mice, whereas **13** was more efficacious at an oral dose of 200 mg/kg. After a continuation of up to 9 days of daily oral administrations with **14** and **15**, further prolongation of cancer survival was revealed without evident loss of body weight.

Compound **14** (BPR0C261, Figure 10) was further examined for its mechanisms behind its anticancer action. It was effectively found to be able to (i) inhibit tubulin polymerization by binding at the colchicine site, (ii) disrupt microtubule arrangement, (iii) arrest the cell cycle at the G2/M phase in cancer cells, and (iv) inhibit angiogenesis in various types of human cancer [79]. In addition, it was orally adsorbable in mice and exhibited good oral bioavailability (43%) in dogs and the ability to cross the human intestinal Caco-2 cell monolayer, suggesting good oral bioavailability in humans. Compound **14** exhibited antiproliferative properties in vivo against different types of tumors, and its combination with cis-platin synergistically prolonged the lifespans of the mice inoculated with murine leukemia cells. Several years later, **14** was shown to be able to modulate the radiation response on human non-small cell lung cancer (NSCLC) cells via p53-dependent and p53-indipendent pathways [80].

**Figure 12:**
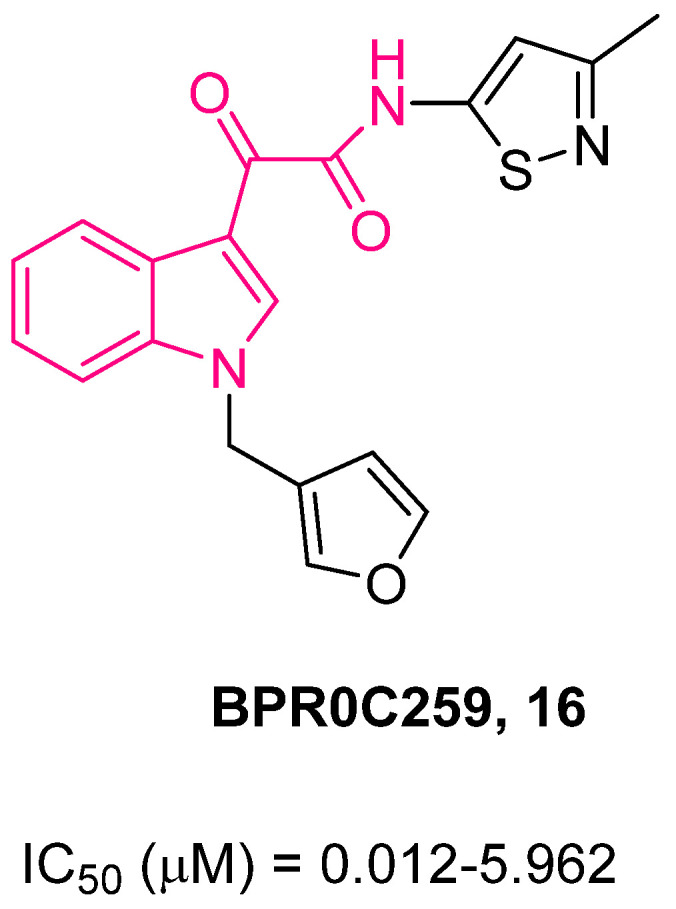
Structure of BPR0C259 **16**.

In this connection, two compounds of this series, **15** and **16**, were assayed on NSCLC cell lines A549 (p53+/+) and H1299 (p53−/−) [81]. Compound **15** (Figure 11) showed a greater cytotoxic effect than **16** (Figure 12) on both cell lines, and this was likely due to its higher cell permeability deriving from its higher lipophilicity. Further biological evaluation highlighted a p53-indipendent antiproliferative mechanism for this class of compounds. Of note, both compounds caused similar patterns of G2/M phase arrest in both cancer cell lines. In addition, they enhanced the radiosensitivity of both cell lines. This effect was probably due to the increased proportion of cells in the G2/M phase, the most radiosensitive cell cycle phase [82].

**Figure 13:**
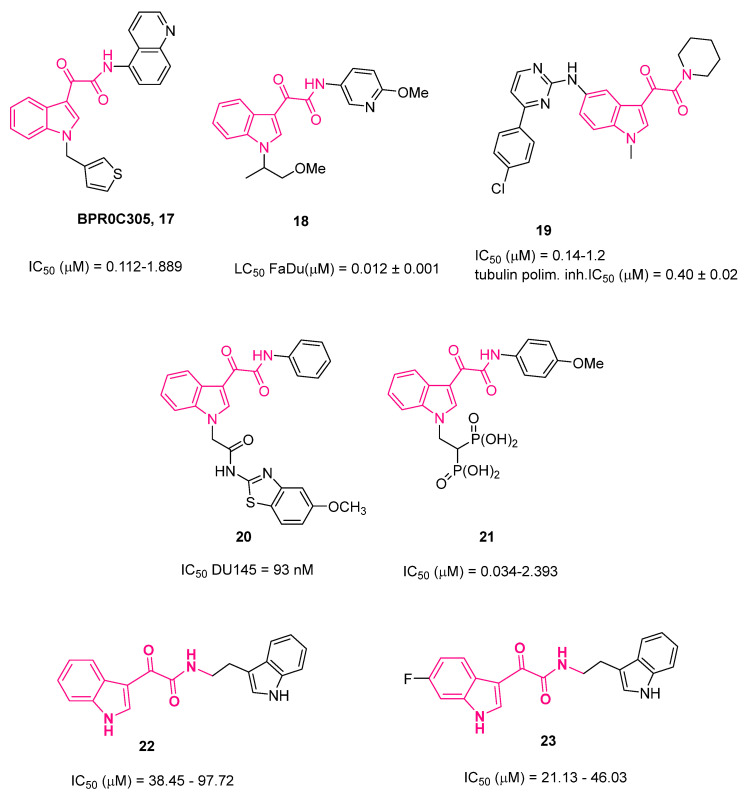
Indol-3-ylglyoxylamides **17**–**23** as antitumor agents.

Pursuing the interest in the development of orally active anticancer drugs, Li et al. showed that compound **17** (BPR0C305, Figure 13) was able to induce cell cycle arrest at the G2/M phase by interfering with microtubule assembly kinetics and to exert antimitotic and antiproliferative effects against human cancer cells. Additionally, **17** is orally adsorbable, is almost safe, and shows anticancer activity against leukemia and solid tumors in mice [83].

Following these studies, Colley et al. [84] in 2015 described a series of **13**-related compounds with less aromatic rings and a greater degree of saturation, aiming to obtain compounds endowed with potent tubulin polymerization activity and improved physicochemical and PK properties. Starting from the suggestion that *N*^1^-unsubstituted indol-3-ylglyoxylamides can still maintain tubulin polymerization inhibitory activity, the authors first focused on the substitution of the *p*-chlorobenzyl group of **13** with an aliphatic moiety. All compounds were initially evaluated for their cytotoxicity against FaDu, an EGFR inhibitor insensitive (head and neck cancer) cell line derived from squamous cell carcinoma of the pharynx. As a general trend, the presence of various simple alkyl groups at the *N*^1^-position induced a loss of cytotoxic activity; however, the introduction of an OCH_3_ group to the pendant pyridine retrieved activity and changing the positions of these structural features caused an improvement in potency. Rational changes in the group at the 3-position (R_2_) revealed some clear trends in activity: (i) the *p*-methoxy group bounded to the pyridine ring played an essential role; (ii) the substitution of the *p*-methoxy with a *p*-ethoxy was tolerated, with a 2-fold increase in LC_50_, whereas the introduction of a second methoxy group was detrimental and (iii) replacement of the pyridine ring with a five-membered heterocycle appeared to be unproductive. The introduction of different groups at the 1-position (R_1_) did led to the desired increase in antiproliferative activity. The introduction of a *sec*-butyl group was more beneficial for the activity compared to the introduction of an isopropyl group. Changing the oxygen atom in the 2-methoxyethyl moiety with a methylene group did not influence activity, suggesting that the shape, rather than the polarity, of the R_1_ group determines the potency. The introduction of an additional α-methyl group to the R_1_ of the 2-methoxyethyl substituent resulted in the most potent compound of the series (**18**, Figure 13, LC_50_ against FaDu = 12 nM) [85], whereas introducing a β-tertiary center was not tolerated at all. In sum, although some of the most potent compounds featured simple and linear R_1_ chains, a degree of further substitution was tolerable or even advantageous, whereas too much substitution led to compounds with severely compromised or abolished cytotoxic activity. Finally, a subset of compounds was further evaluated, and it was discovered that they were effectively able to inhibit tubulin polymerization through binding at the colchicine site.

Guggilapu and coworkers developed a small library of C5-tethered indol-3-ylglyoxylamides as tubulin polymerization inhibitors [86]. All derivatives were tested for cytotoxic activity against different cancer cell lines (prostate (DU145, PC-3), lung (A549), and colon (HCT-15)), with the DU145 cell line highlighted as the most sensitive one. In particular, derivative **19** (Figure 13) was shown to be the most cytotoxic one and was further biologically evaluated, evidencing its ability to (i) inhibit cell migration, (ii) induce apoptosis, (iii) arrest the cell cycle at the G2/M phase in a dose-dependent manner, (iv) inhibit tubulin polymerization with an IC_50_ value of 0.40 μM, (v) induce the collapse of mitochondrial membrane potential (Δψm), (vi) increase ROS levels, and (vii) decrease anti-apoptotic Bcl-2 and increase pro-apoptotic Bax levels.

In a subsequent study, the same research group developed a class of compounds by merging the indol-3-ylglyoxylamide scaffold with the bioactive thiazole nucleus [87]. All compounds were first evaluated for antiproliferative activity against different cancer cell lines (prostate (DU145, PC-3), lung (A549), and colon (HCT-15)) and, also for this class, the DU145 cell line turned out to be the most sensitive one. The most active compound was **20**, which showed IC_50_ = 93 nM against DU145, was safe with the healthy cell line RWPE-1, and inhibited tubulin polymerization with a percentage of 68.5%. Moreover, DU145 treatment with compound **20** led to (i) cell migration inhibition, (ii) apoptosis induction, (iii) G2/M phase cell cycle arrest in a concentration-dependent manner, (iv) the collapse of Δψm, and (v) an increase in ROS levels. Finally, molecular modeling studies performed on **20** evidenced the crucial role played by the glyoxyl moiety, which was able to establish three hydrogen bonds with active site residues of the colchicine binding site of the tubulin [87].

More recently [88], aiming at developing more water-soluble compounds, Brel and colleagues synthesized indibulin **13** (Figure 9) derivatives featuring a bisphosphonate moiety on the indole nitrogen. Assessment of the compounds’ antimitotic microtubule-destabilizing activity and cytotoxicity toward a panel of human cancer cells outlined the tetraacid derivative **21** (Figure 9) as the most active compound, which was more potent with respect to indibulin **13**. The storage of stock solutions of its corresponding tetraethyl esters in 96% EtOH at room temperature provoked spontaneous hydrolysis of bisphosphonate and subsequent time-dependent activity enhancement. Compound **21** also showed significant cytotoxicity toward A549 and A375 human cancer cells. At physiological pH, **21** formed tetrasodium salt endowed with an aqueous solubility of at least 10 mg/mL, three orders of magnitude higher than that of indibulin **13**.

In 2016, Tantak’s research group [89], encouraged by the good antitumoral activity shown by bisindole-based derivatives and by the crucial role played by the glyoxylamide in bioactive compounds [90,91], synthesized and tested a series of bis(indolyl)glyoxylamides against different human cancer cells. The most promising compounds, **22** and **23**, are presented in Figure 13. Studies on the preliminary mechanism of action indicated the ability of these compounds to induce apoptosis in PC-3 cells by increasing cleaved PARP1 levels.

**Figure 14:**
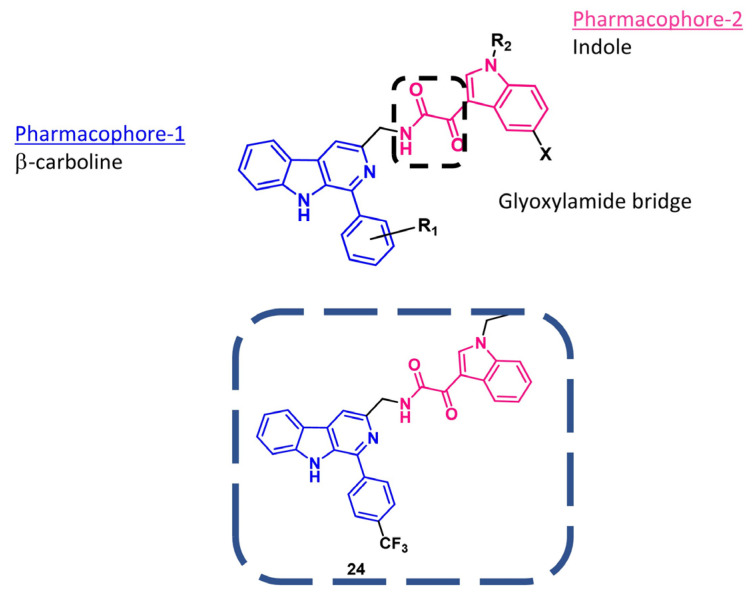
Design of β-carboline indol-3-yl-glyoxylamide hybrids as antitumor agents.

Very recently, Soni et al. [92] designed and synthesized a series of compounds by joining the indol-3-ylglyoxylamide moiety and the β-carboline scaffold to obtain cytotoxic agents acting as DNA intercalators. In particular, the β-carboline alkaloid skeleton was incorporated as a pharmacophoric planar aromatic ring for DNA intercalation [93,94] and the indole ring was introduced as a second pharmacophoric requirement of the hybrids. Lastly, the glyoxamide may have helped the molecule to assume the appropriate orientation in the active site (Figure 14).

When tested for their antiproliferative effects toward several human cancer cell lines, the most potent compound was **24** (Figure 14), with IC_50_ values ranging from 4.37 to 10.36 μM and good selectivity to cancer cells with respect to healthy ones. Deep biological evaluation revealed that **24** was able to (i) induce early and late apoptosis, (ii) bind DNA, and (iii) inhibit topoisomerases II (TopoII). The interaction of **24** with a DNA-TopoII complex was also studied in silico by molecular modeling analysis, revealing the crucial role of glyoxylamide moiety, which is implicated in a hydrogen bond with DNA. An in silico study also revealed promising drug-like properties of **24**.

**Figure 15:**
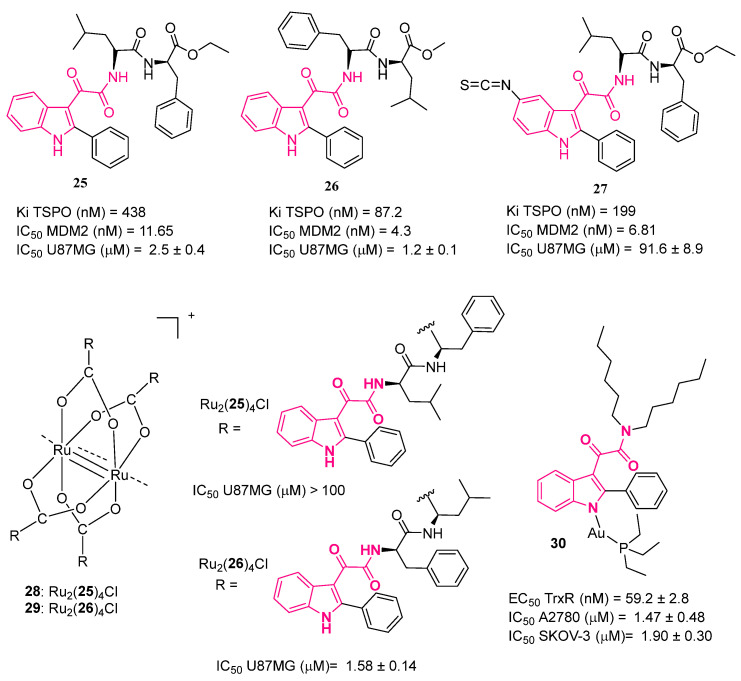
Indol-3-ylglyoxylamides **25**–**30** as antitumor agents.

The indol-3-ylglyoxylamide scaffold was exploited by Taliani et al. to identify compounds useful for the multi-target treatment of glioblastoma multiforme (GBM), a particularly aggressive form of brain cancer. In fact, the pathogenesis of malignant gliomas was characterized by the deregulation of different intracellular signaling pathways, most of them converging to escape from cell death, which is a common feature of tumors and the main cause of treatment failure [95]. In this respect, apoptosis inducers have emerged as promising drugs for a large variety of tumors. In GBM, tumor suppressor protein p53 [96] and mitochondrial 18 kDa translocator protein (TSPO) [97] represent two attractive intracellular targets, both acting as apoptosis inducers. On this basis, indol-3-ylglyoxyldipeptides were rationally designed to dually modulate TSPO and p53 [98]. Specifically, the authors focused their attention on their in-house collection of 2-phenylindol-3-ylglyoxylamide TSPO ligands (PIGAs) [47,48,49], which were suitable to be easily structurally modified, and, aided by molecular modeling studies, planned adequate decoration of the basic scaffold, with the aim of reactivating p53 while retaining TSPO binding affinity. The result of the study was the identification of compound **25** (Figure 15), which was able to bind TSPO (Ki = 438 nM) and reactivate p53 functionality by inhibiting (IC_50_ = 11.65 nM) the dissociation from its physiological inhibitor, murine double minute 2 (MDM2) [98]. Compound **25** was also able to induce Δψm collapse and inhibit cell viability in GBM cells. Of note, **25** was more active with respect to the single target reference standards applied alone, nutlin-3 for MDM2-p53 and PK11195 for TSPO, and was as potent as the combination of them. This is consistent with the synergism resulting from the simultaneous targeting of TSPO and p53, which may represent a valuable anticancer therapy to treat GBM, since most GBM phenotypes maintain wild-type p53 and over-express TSPO and MDM2 [98].

Encouraged by these good results, the same research group started a lead optimization process and developed a small library of 2-phenylindol-3-ylglyoxylyldipeptide derivatives in their methyl ester form [99].

From this study, compound **26** (Figure 15) emerged as the most active compound in terms of TSPO binding affinity and p53-MDM2 interaction inhibition (TSPO: Ki = 87.2 nM, p53-MDM2: IC_50_ = 4.3 nM) [99]. Moreover, its effects were superior to those of the lead, **25**. For these reasons, it was selected for further studies aimed at characterizing its biological effectiveness as an antitumoral agent. The results evidenced the ability of **26** to restore normal p53 activity and inhibit GBM cell growth by cell cycle arrest and apoptosis. Furthermore, **26** was ineffective at affecting the viability of a GBM cell line expressing mutant p53, whereas it was able to reduce the proliferation of glioma cancer stem cells, cells within the tumor that are resistant to therapies and responsible for GBM recurrence. Cell viability assays on non-tumoral human mesenchymal stem cells (MSCs) evidenced the ability of **26** to display its effect predominantly on tumor cells with respect to healthy ones [99].

In cancer therapy, reversible drugs may have several limitations, including the lack of ability to induce a therapeutic effect over time and the consequent activation of alternative signaling pathways that escape drug action, causing resistance [100]. In this connection, Taliani et al. [101] developed a dual target compound able to covalently bind MDM2 and TSPO (**27**, Figure 15). In detail, starting from the previously developed reversible derivative **25**, the authors introduced the chemo-reactive isothiocyanate group at the 5-position of the indole ring, a position that is not implicated in the interaction with the target protein [98]. Then the ability of compound **27** to bind TSPO with a long-lasting binding profile was shown (Ki = 199 nM). Through this binding, **27** caused mitochondrial permeability transition pore opening and consequent Δψm dissipation in GBM cells. Both effects were shown to be maintained over time, unlike those observed for the reversible analogue **25**. In addition, **27** bound MDM2 with an IC_50_ of 6.81 nM, inhibiting its interaction with p53; also in this case, the effect was sustained over time, thus demonstrating the covalent nature of such an interaction. Compound **27** was also able to inhibit GBM cell growth by arresting the cell cycle and inducing apoptosis. All of these effects appeared to be greater and more long lasting with respect to those elicited by **25**. Moreover, the apoptosis appeared to be irreversible, hindering the cells from recovering proliferative activity after the drug was washed out. Overall, all these findings demonstrated that in GBM cells compound **27** acts as a dual-targeting and irreversible ligand, representing a useful alternative to overcome the time-limited effects of classical chemotherapies [101].

Platinum-based agents, i.e., cisplatin, carboplatin, and oxaliplatin, are some of the most used and active chemotherapeutic drugs currently prescribed to treat many types of human cancer [102]. However, behind their effectiveness, their use is limited by drug resistance and adverse effects [103]. Accordingly, several effective strategies, including the use of transition metals different from platinum, have been developed [104]. Among these, paddlewheel ruthenium-based complexes, featuring a direct metal–metal bond and a (II,III) mixed valence, emerged as very promising [105]. In this regard, a diruthenium (II,III) complex **28** (Ru_2_(**25**)_4_Cl) (Figure 15) was developed by reacting Ru_2_(μ-O_2_CCH_3_)_4_Cl with the dual agent **25** [98,106]. At variance with **25**, **28** was ineffective in GBM because of its high stability, which is due to the steric hindrance provided by indolylglyoxylyl moiety that hampers the attack of the water molecules or other potential ligands at the Ru metal centers, inhibiting the release of ligand and, in turn, its anticancer effect. Thus, complex **29** (Ru_2_(**26**)_4_Cl) (Figure 15) was developed, featuring **26** (isomer of **25**) [99], which coordinates the Ru_2_ core. Complex **29** was less stable and, in turn, more active as an antitumoral agent due to the enhanced availability of the Ru_2_ core to attack by nucleophiles with respect to **28** [107].

The same research group in 2023 developed a complex designed as an auranofin analogue obtained by conjugating a PIGA ligand [47,48,49] with the auranofin-derived cationic fragment [Au(PEt_3_)]^+^ [108]. Auranofin is a gold-based drug clinically used for the treatment of arthritis. Lastly, it took part in different drug repurposing strategies, and it was shown to be promising toward different tumors, such as ovarian cancer (OC), in particular by inhibiting thioredoxin reductase (TrxR), localized at the mitochondrial level [109]. In parallel, TSPO expression was altered in various multiple diseases, including OC [110]. In view of this, the rationale of the design is to exploit the high TSPO affinity of phenylindolylglyoxylamide moiety to drive the complex into the mitochondria, where the [Au(PEt_3_)]^+^ cation, after its release, can exert its anticancer function. Notably, complex **30** (Figure 15) exhibited enhanced antitumor activity toward two models of human OCs (i.e., A2780 line and SKOV-3) and much lower cytotoxicity toward a healthy cell line (i.e., HEK293). In addition, **30** was effective as a TrxR inhibitor, with EC_50_ in the low nanomolar range.

## 5. Indolylglyoxylamides with Antibacterial Properties

**Figure 16:**
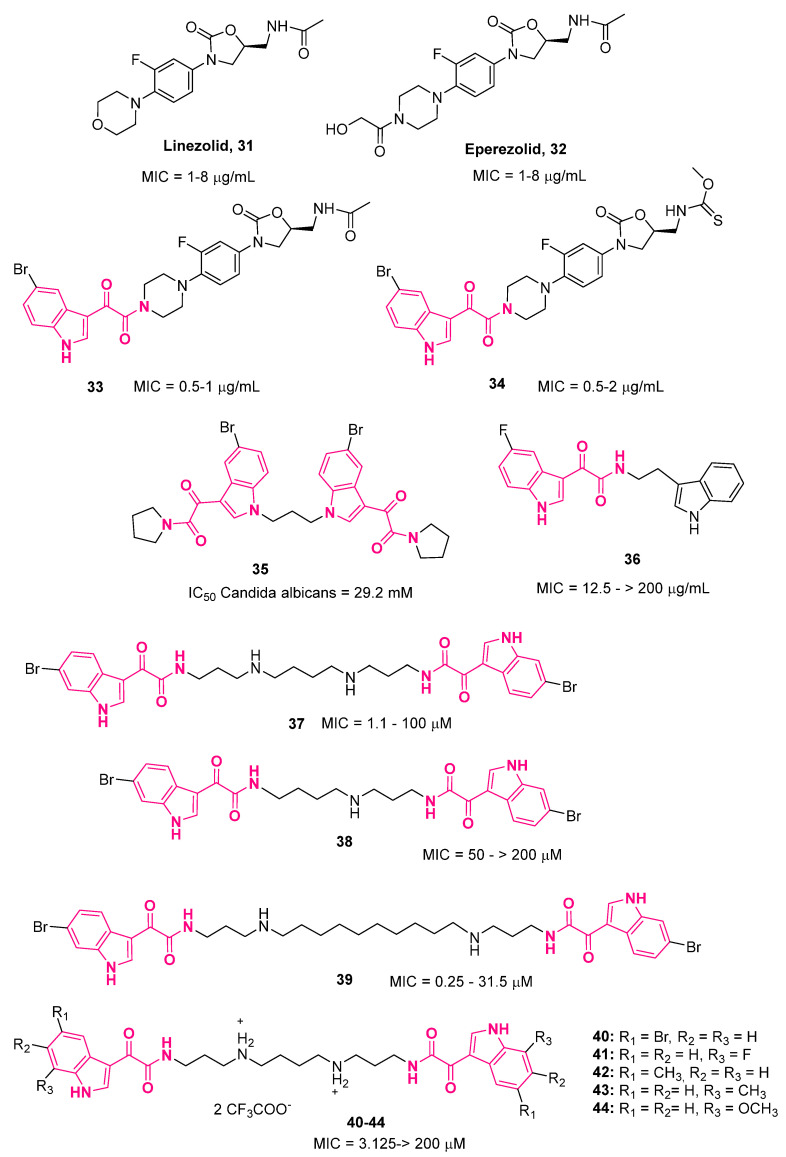
Linezolid **31**, eperezolid **32**, and indol-3-ylglyoxylamides **33**–**44** as antibacterial agents.

In 2008, Takhi and colleagues [111] developed a small library of 3-indolylglyoxylamides endowed with antibacterial properties structurally related to linezolid **31** and eperezolid **32** (Figure 16), oxazolidinone-based antibacterial agents effective against many susceptible and resistant Gram-positive organisms such as methicillin-resistant *Staphylococcus aureus* (MRSA), vancomycin-resistant enterococci, and penicillin-resistant *Streptococcus Pneumoniae* (PRSP) [112]. The oxazolidinones inhibited bacterial protein synthesis by binding to a site on the 50S ribosomal subunit, thus preventing the formation of a functional 70S initiation complex [113,114]. Despite that, with this single mechanism, no cross-resistance was expected between this class and other antibacterial families, although some cases of **31**-resistant pathogens in clinical have been observed [115]. In this context, to increase the spectrum and potency of these oxazolidinones, Takhi’s research group exploited the indolylglyoxylamide moiety. Two series of compounds were synthesized: the first one by replacing the hydroxyacetamido group of **32** with various substituted 3-indolylglyoxylamides, and the second one by replacing the acetamide at the *C*-5 position with a variety of *N*- and *O*-linked groups and keeping the 5-bromoindole fixed on the left side. Almost all derivatives showed increased activity with respect to **31** against MRSA, *Enterococcus faecalis* (*E. faecalis*), and *Enterococcus faecium* (*E. faecium*). Among these compounds, the most active one was 5-bromoindole **33** (Figure 16), which showed 1-2-fold higher activity with respect to **31** against a panel of organisms. The MIC values against *Staphylococcus aureus* (*S. aureus*)*, E. faecalis*, and *E. faecium* were in the range of 0.5–1 μg/mL. However, all derivatives were ineffective across a Gram-negative community acquired pneumonia pathogen-like *Haemophilus influenzae* (*H. influenzae*) [111].

In a subsequent phase, starting from **33**, a class of molecules was developed in which the 5-bromoindole moiety was kept fixed, whereas the acetoamidomethyl side chain was replaced with various substituents, such as amide, carbamate, and azole. In general, almost all compounds were shown to be active across all the organisms investigated, except for the Gram-negative *H. influenzae*, as observed for the first series. *N*-linkage modifications at *C*-5 furnished the most active compounds of the series, such as **34** (Figure 16) (MIC = 0.5–2 μg/mL), which also demonstrated in vivo efficacy against *S. aureus* DRCC035 in a lethal murine infection model via oral route.

Singh and colleagues developed a series of *N*-1, *C*-3, and *C*-5 substituted bis-indoles [116], among which **35** (Figure 16) was the most active compound, being able to generate the highest zone of inhibition. Molecular modeling studies performed on the enzyme targeted by clinically used antimicrobial agents (TopoII, dihydrofolate reductase) evidenced that the glyoxylamide moiety of **35** plays a key role in such interactions [116].

In 2016, Tantak’s research group also made use of the indol-3-yl-glyoxylamide scaffold to design potential antibacterial agents [89]. In detail, encouraged by the good antimicrobial activity of bisindole-based derivatives [116,117], a series of bis(indolyl)glyoxylamides was synthesized and tested against two Gram-positive bacteria, *Bacillus subtilis* and *S. aureus*, and three Gram-negative bacteria, *Escherichia coli* (*E. coli*), *Pseudomonas putida*, and *Klebsiella pneumonia (K. pneumoniae*). The most promising compound was **36** (Figure 16), which showed significant antibacterial activity across all the investigated organisms, with MIC values ranging from 12.5 to 25 μg/mL, combined with high effectiveness in killing Gram-negative organisms.

Via a screening campaign on compounds from an in-house library, Li et al. [118] identified two 6-bromoindol-3-ylglyoxylamido-polyamines, **37** and **38** (Figure 16), which displayed antimicrobial activity across Gram-positive bacteria *Staphylococcus intermedius* (MIC 3.125 µM for both compounds) and *S. aureus* (MIC 6.25 and 3.125 µM, respectively), with **37** also being able to enhance the in vitro antibiotic activity of doxycycline toward the resistant Gram-negative *Pseudomonas aeruginosa* (*P. aeruginosa*). Starting from these results, a series of 6-bromoindol-3-ylglyoxylamido-polyamines was developed, leading to the discovery of compounds with enhanced antimicrobial activity. Compounds with a polyamine (PA) core were the most active within the series, reaching maximal potency with a PA3-10-3 core (**39**, Figure 16). Compound **39** also displayed the ability to enhance the antibiotic activity of doxycycline toward *P. aeruginosa*. Deep investigation of **37** revealed its ability to (i) restore antibiotic activity against a variety of Gram-negative bacteria, such as *Escherichia coli* (*E. coli*), *K. pneumoniae*, and *Acinetobacter baumannii* (*A. baumannii*); (ii) disrupt bacteria membrane integrity; and (iii) inhibit the efflux pump via depolarization of the membrane [119]. Unfortunately, **37** exhibited cytotoxicity and strong red blood cell hemolytic activity.

In the same year [120], the same researchers, using **37** as a lead compound, described a series of bromoindol-3-ylglyoxylamides in search of antibacterial agents lacking cytotoxic/hemolytic activity. All the compounds showed intrinsic activity across Gram-positive bacteria and the fungus *Cryptococcus neoformans*, with **40** (Figure 16) also displaying slight activity across Gram-negative bacteria like *E. coli* (MIC 25 µM) and *K. pneumoniae* (MIC 34.4 µM). Most derivatives also potentiated the activity of doxycycline against Gram-negative bacteria such as *P. aeruginosa*, *A. baumannii*, *E. coli*, and *K. pneumoniae*. This study allowed the authors to identify five antibacterial compounds (**40**–**44**, Figure 16) without cytotoxic/hemolytic activity. Their mechanism of action consisted of disrupting the integrity of the bacterial membrane and inhibiting the efflux pump by means of depolarization of the membrane, as observed for the lead, **37**.

## 6. Indolylglyoxylamides with Anti-Inflammatory Properties

### 6.1. Phosphodiesterase PDE4 Inhibitors

Phosphodiesterases (PDEs) are enzymes responsible for the modulation of signal transduction, and they catalyze the degradation of cyclic nucleotides (cAMP and/or cGMP). Among the 11 families known, PDE4, PDE7, and PDE8 are selective for cAMP [121]. As the PDE4 isozyme is the most prominent cAMP metabolizing enzyme in immune and inflammatory cells, selective PDE4 inhibitors have been deeply investigated, with the aim of developing novel anti-inflammatory agents alternative to corticosteroids [122]. Potent anti-inflammatory effects in patients with inflammatory diseases such as asthma or chronic obstructive pulmonary disease (COPD) were observed following selective inhibition of PDE4 [123].

PDE4 inhibitors are more potent than corticosteroids in clinical use because they can influence diverse cell types involved in inflammatory diseases, including respiratory epithelial cells, smooth muscle cells, and submucosal glands. Furthermore, they lack the common side effects associated with corticosteroid therapy, namely, adverse reactions on the pituitary–hypophyseal axis and on bone density, among others [124]. However, the therapeutic use of PDE4 inhibitors so far has been limited by several side effects, such as nausea, vomiting, and headache.

**Figure 17:**
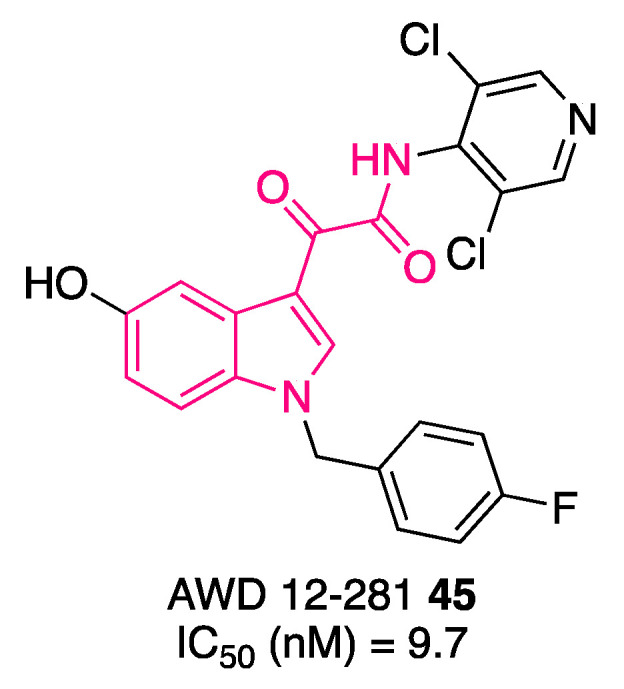
Structure of indolylglyoxylamide PDE4 inhibitor **45**.

Compound **45**, AWD12-281 (Figure 17), was a highly selective PDE4 inhibitor [125,126] with a better safety profile than other PDE4 inhibitors, namely cilomilast and roflumilast [127,128], in clinical development. Additionally, it was demonstrated that when **45** was administered topically by inhalation in dogs, also at the highest suitable dose (15 mg/kg in 50% lactose blend), no emesis was produced. These data support its potential use for the topical treatment of asthma and COPD. It is characterized by low oral bioavailability and solubility, and it exerts robust and durable pharmacological effects after intratracheal administration in various animal models, indicating persistence in lung tissue. Compound **45** was capable of suppressing the production of cytokines in stimulated peripheral blood mononuclear cells (PBMCs): interleukin-2 (IL-2, phytohemagglutinin stimulation), IL-5 and IL-4 (anti-CD3/anti-CD28 costimulation), and lipopolysaccharide-stimulated release of tumor necrosis factor α (TNFα). The corresponding EC_50s_ for **45** were within a narrow range (46–121 nM). Compound **45** also suppressed TNFα release in dispersed nasal polyps (EC_50_ = 111 nM) and in diluted whole blood (EC_50_ = 934 nM). High plasma protein binding may be responsible for the reduced activity in human blood. The molecule entered phase II clinical trials to evaluate its therapeutic potential in asthma, COPD, and allergic rhinitis. Even though the results are not available to the public, development was discontinued in 2006 due to poor efficacy [129]. Despite the halt, the good safety profile, along with the persistence in lung tissue, may serve as a good starting point for the development of novel clinical candidates for the aforementioned pathological conditions. 

### 6.2. Phospholipase A2 Inhibitors

Phospholipases A_2_ (PLA_2_s) belong to the superfamily of phospholipases, enzymes that hydrolyze phospholipids. PLA_2_s are lipolytic enzymes responsible for the catalysis of the ester bond hydrolysis at the sn-2 position of glycerophospholipids, which generate free fatty acids, including arachidonic acid and lysophospholipids. This family comprises four predominant types of PLA_2_: secreted PLA_2_ (sPLA_2_), cytosolic Ca^2+^-dependent PLA_2_ (cPLA_2_), cytosolic Ca^2+^-independent PLA_2_ (iPLA_2_), and PAF-AH (platelet activating factor acetyl hydrolases). The other two types are lysosomal PLA_2_ (LPLA_2_) and adipose-PLA_2_ (AdPLA). Enzymatic functions are performed by means of a catalytic dyad/triad (His/Asp for sPLA_2_; Ser/Asp for cPLA_2_ and iPLA_2_; Ser/His/Asp for PAF-AH and LPLA_2_; His/Cys for AdPLA) [130].

sPLA_2_s are involved in several inflammatory diseases. The first indication came from high levels of group IIA (GIIA) sPLA_2_ found in the synovial fluid of patients with rheumatoid arthritis. Abnormal levels of sPLA_2_s were also found in the plasma or serum of patients with acute pancreatitis, septic shock, Crohn’s disease, and ulcerative colitis. Furthermore, sPLA_2_s seem to be involved in adult respiratory distress syndrome and inflammatory bowel disease [130]. 

**Figure 18:**
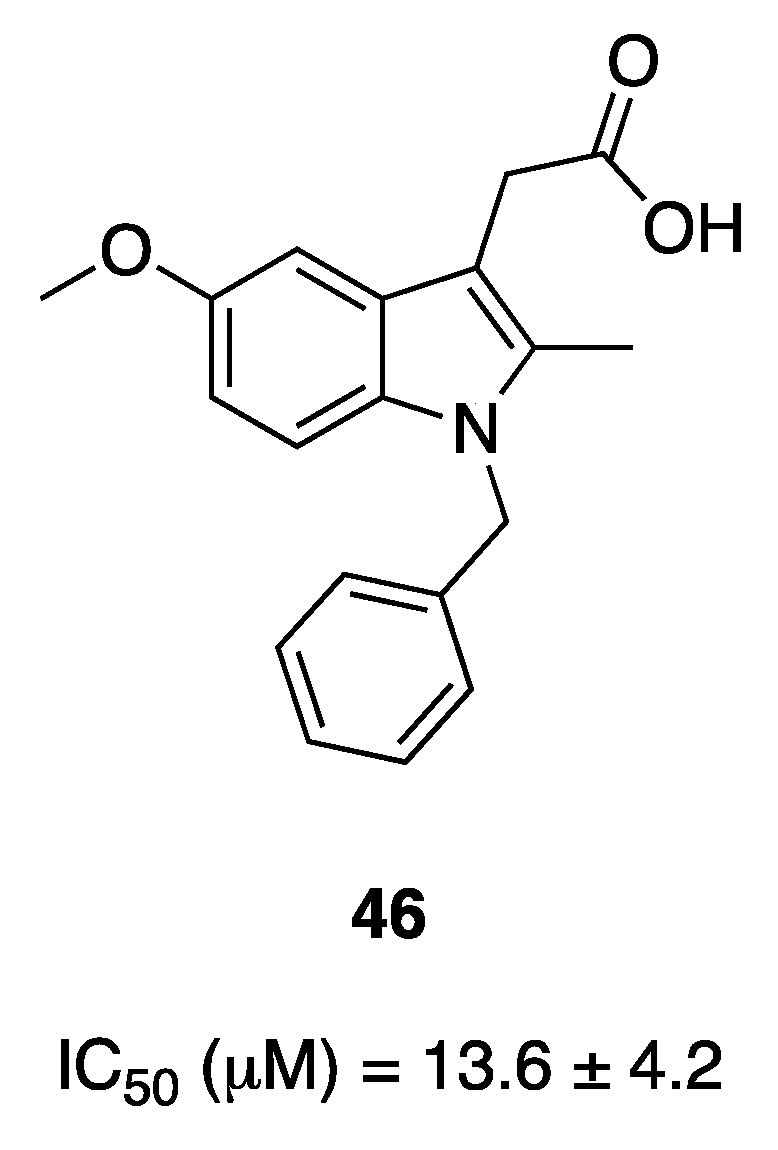
Structure of the first indole derivative synthesized by Lilly as sPLA_2_ inhibitor **46** [131].

Researchers at Lilly published a series of papers regarding indole-based derivatives as inhibitors of GIIA sPLA_2_ (referred to by the authors as “human nonpancreatic secretory phospholipase A_2_,” hnps-PLA_2_) [131]. Lead compound **46** (IC_50_ = 13.6 μM, Figure 18) was initially obtained by high-volume screening, and several rounds of optimization were performed, including the replacement of the acetate function first with an acetamide moiety and then with the α-ketoamide group [132].

Incorporation of the α-ketoamide group, yielding the indolylglyoxylamide moiety, was crucial, as delineated by compounds **47**–**50** (Table 2), in which the introduction of a 4-oxyacetic acid group allowed optimal potency and selectivity to be reached [133].

Interactions of the lead compound **46** were confirmed by X-ray crystallography studies, which also allowed for a rationalization of the effective binding between the calcium ion in the active site of hGIIA and the carbonyl of the 4-substituent and the carboxamide carbonyl of the 3-glyoxylamide moiety of compound **50** (LY315920, varespladib) [134]. Introduction of the glyoxylamide moiety was instrumental to additional interactions in the active site, namely, the hydrogen bond formation between the carboxamide and His 48, and the new interaction between the ketone carbonyl and Phe 106 of the enzyme [133]. 

Several clinical trials were launched to evaluate the efficacy of Varespladib **50**, also formulated as a methyl ester prodrug, for various diseases (i.e., sepsis-induced systemic inflammatory response syndrome, asthma, cardiovascular diseases), but the molecule did not demonstrate sufficient efficacy in either phase II or phase III [135,136,137,138,139,140,141]. 

### 6.3. p38α Inhibitors

p38α belongs to the well-known mitogen-activated protein (MAP) kinase family of serine/threonine protein kinases. It is widely expressed in endothelial, immune, and inflammatory cells and plays a pivotal role in the release of proinflammatory cytokines such as TNF-R, IL-1β, and IL-6 [142,143]. Compounds able to selectively block any one of these cytokines have proven efficacious for the treatment of pathological inflammatory conditions, including rheumatoid arthritis (RA), psoriasis, and inflammatory bowel disease [144]. Separate genes encode for the four isoforms (p38α, p38β, p38γ, and p38δ) of the p38 subfamily of MAP kinases. The p38α isoform was overactivated within inflamed tissues, which suggests that this target could be used for the treatment of these types of diseases [145,146].

Starting from the ability of p38α inhibitors to block the synthesis and release of proinflammatory cytokines, several pharmaceutical companies, like Merck, Vertex, Roche, and Scios (subsidiary of J&J), among others, invested in the development of novel therapeutic agents for the treatment of RA, inflammatory bowel disease, psoriasis, systemic lupus erythematosus, and other indications characterized by chronic inflammation. This led to the development of an impressive variety of chemically diverse competitive inhibitors with excellent drug-like properties. These compounds showed a high degree of selectivity despite most of them being ATP competitive inhibitors. This is because these compounds, although structurally different, position themselves in specific regions in or in proximity to the ATP binding site of p38α, which is characterized by amino acid sequences distinct from the majority of other human kinases. Being able to identify such selectivity hotspots is an important process to developing highly selective ATP competitive kinase inhibitors.

**Figure 19:**
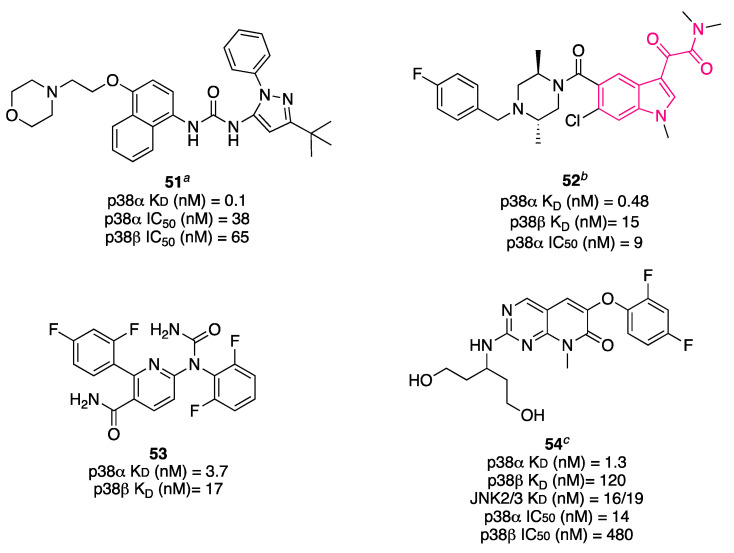
Structure of derivatives **51**–**54** as p38α inhibitors. *^a^* IC_50_ values taken from Kuma et al. [151]. *^b^* IC_50_ value taken from Goldstein et al. [152]. *^c^* IC_50_ values taken from Hill et al. [150].

First- and second-generation p38α inhibitors, like **51** (Figure 19) [147], did not show high selectivity against other human kinases. However, third-generation inhibitors have recently been developed, demonstrating that selective p38α inhibition is possible. Three third-generation compounds, **52** [148], **53** [149], and **54** [150] (Figure 19), are highly kinase selective.

In a panel of 321 human protein kinases, Scios inhibitor **52** bound exclusively to p38α (K_D_ = 0.48 nM) and its closely related isoform p38β (K_D_ = 15 nM). Vertex inhibitor **53** bound to p38α (K_D_ = 3.7 nM), p38β (K_D_ = 17 nM) and five other kinases. Roche inhibitor **54** bound to p38α (K_D_ = 1.3 nM) and to JNK2/3 and p38β, although less strongly (K_D_ =16/19 nM and K_D_ = 120 nM, respectively); in addition, it bound weakly to five other kinases [152]. Compound **52** displayed an IC_50_ = 9 nM against p38α. It was also able to inhibit LPS-induced TNFα release from human whole blood (IC_50_ ≈ 0.3 μM) and LPS-induced IL-1β release from human peripheral blood mononuclear cells (PBMCs) in a concentration-dependent manner [153]. A phase I study evaluted the safety, pharmacodynamics, and PK of **52** in healthy volunteers. A phase II study investigated the analgesic efficacy of **52** in acute postsurgical dental pain (263 subjects). Because it is well known that inflammatory mediators and cytokines such as TNFα and IL-1 can contribute to peripheral and central sensitization and also to modulating acute, chronic, and neuropathic pain, inhibition of p38 could be beneficial to managing pain [154,155]. This study showed for the first time the acute analgesic effects induced by inhibition of p38α. The molecule was evaluated in a phase II clinical trial in patients with active rheumatoid arthritis and showed no greater efficacy compared to the placebo [156]. 

Recently, a combination of compound **52** with remdesivir has been suggested to treat stage I, stage II, or stage III COVID-19 or COVID-19 cytokine storm [157].

## 7. Indolylglyoxylamides with Antiviral Properties

### 7.1. Viral NS2B/NS3 Protease Inhibitors

Dengue virus (DenV) is the infectious agent responsible for Dengue fever, a neglected tropical disease. It belongs to the Flaviviridae family, a family of viruses characterized by a positive single-stranded RNA genome [158,159]. Essential to the viral replication cycle is the NS2B/NS3 protease, a serine endoprotease member of the chymotrypsin family expressing the catalytic triad His51–Asp75–Ser135. The protease cleaves the polyprotein encoded by the viral genome into functional proteins, thus representing an interesting therapeutic target [160].

**Figure 20:**
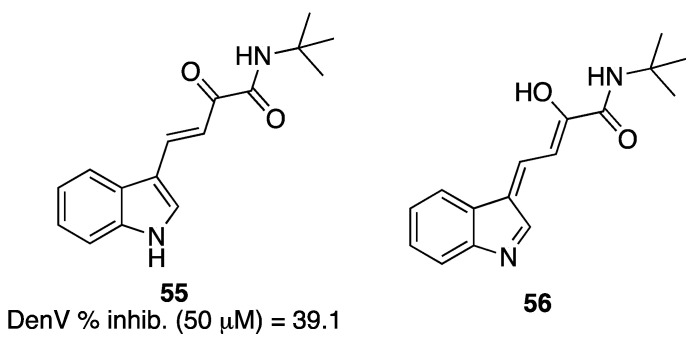
Structure and biological activity of derivative **55** and its possible tautomer, **56**.

Klein and colleagues [161] synthesized several β,γ-unsaturated-α-ketoamides, and among these compounds, the most interesting derivative, **55** (Figure 20), was able to inhibit DenV replication in a cell-culture assay in a concentration-dependent manner and to induce a 1000-fold reduction in virus titer at noncytotoxic concentrations. SARs highlighted the role of the indolylglyoxylamide moiety, with the α-ketoamide warhead showing superior inhibitory activity compared to α-hydroxy and α-epoxy derivatives, and the indole portion binding to the S1 subsite. It should be speculated that **55** may tautomerize to **56** because of the double bond at the α,β-position with respect to the ketocarbonyl (Figure 20) and that both may contribute to the activity of the compound.

Zika virus (ZIKV) is another pathogen belonging to the Flaviviridae family and Flavivirus genus, like DenV. ZIKV infections are usually asymptomatic or characterized by mild symptoms, but during the latest outbreaks severe ZIKV-associated complications emerged (depending on the genetic variant of ZIKV), such as congenital birth defects, including microcephaly, and Guillain–Barre syndrome in adults [162]. In 2018, ZIKV infection was added to the Research and Development Blueprint by the WHO (the list of diseases recommended for intensive research) [163]. 

**Figure 21:**
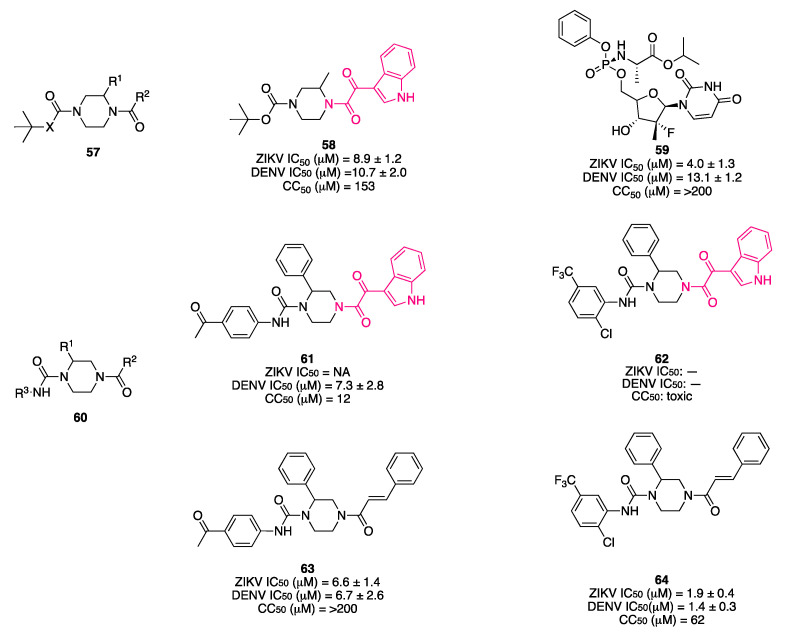
General structure of the first series of acyl piperazine amide and urea derivatives **57** and of optimized urea derivatives **60** and the biological activity of **58**, sofosbuvir **59**, indolylglyoxylamides **61**–**62**, and cinnamoyl derivatives **63**–**64** as antiviral agents [164].

Del Rosario García-Lozano et al. investigated a series of piperazine-based small molecules to find inhibitors with broad activity against Flaviviridae [164]. By means of a privileged structure-based design, the authors coupled the central piperazine core with preferred medicinal chemistry structures, which has proven to possess multiple biological activities, namely, indol-3-yl-2-oxoacetyl [11,161,165,166,167], cinnamoyl [168,169,170,171,172,173,174,175,176], and quinoline-3-carbonyl groups [177,178,179,180,181,182,183,184]. They initially synthesized 26 acylpiperazine amide and urea derivatives (general formula **57**, Figure 21), which were screened by a commercial HCV NS3/4A protease inhibition assay, resulting in inhibitory values above 70%, and then assessed in a cell-based antiviral assay to determine their potential activity to inhibit ZIKV and DenV replication in vitro. From the first round of testing, compound **58** (Figure 21), featuring an indolylglyoxylamide, emerged as an interesting molecule with dual activity against ZIKV (IC_50_ = 8.9 μM) and DenV (IC_50_ = 10.7 μM) and low toxicity (CC_50_ = 153 μM). Antiviral activity of this molecule is comparable to the protease inhibitor sofosbuvir **59** (ZIKV IC_50_ = 4.0 μM, DENV IC_50_ = 13.1 μM, CC_50_ > 200 μM, Figure 21), which was used as a reference compound.

In a subsequent round of optimization, the urea linker was functionalized with various aromatic substituents (general formula **60**, Figure 21). In this second set of compounds, the indolylglyoxylamide derivatives **61** and **62** (Figure 21) performed worse than the cinnamoyl ones, **63** and **64** (Figure 21). Compound **61** showed activity only against DenV (IC_50_ = 7.3 μM), and the toxicity profile was not optimal (CC_50_ = 12 μM), whereas compound **62** was just toxic. Compound **64** was the best in terms of activity against ZIKV (IC_50_ = 1.9 μM) and DenV (IC_50_ = 1.4 μM), showing values lower than sofosbuvir **59**. Although compound **64** was the most potent of the study, compound **58** showed a lower LogP value (2.17 vs. 5.15) and a minor fraction bound to plasma protein (78.00% vs. 91.97%) in the predicted ADME properties, confirming that the properties of the indolylglyoxylamide moiety are interesting for drug development.

### 7.2. HIV-1 Inhibitors

The human immunodeficiency virus (HIV) infection pandemic is now over 30 years old and continues to be a global health issue, with more than 37 million infected people (https://www.unaids.org/en/resources/fact-sheet, accessed on 10 June 2023). The utilization of highly active antiretroviral therapy (HAART) helped to impede the spread of HIV and transformed it into a chronic disease for many patients. However, continuous treatment with HAART can lead to limitations, such as the onset of drug resistance. In addition, for this reason, there is still a need for the development of new anti-retroviral agents that can target different steps of the replication cycle with improved tolerability and dosing schedules [185]. Among the numerous steps of the HIV infection cycle, the specific interaction between the membrane-bound HIV-1 glycoprotein 120 (gp120) and cluster of differentiation 4 (CD4), the primary attachment receptor for HIV-1, represents an attractive target for the development of novel inhibitors since the disruption of this interaction would likely curb HIV-1’s infectivity at a very early stage of the viral infection [186].

In this regard, a cell-based screening assay led to the identification of indolylglyoxylamide derivative **65** (Table 3), which was demonstrated to interfere with the gp120/CD4 interaction. Subsequent optimization studies on **65** generated molecules strongly able to inhibit HIV-1 infection in vitro [187,188,189,190], including indolylglyoxylamide **66** (Table 3), which possessed nanomolar EC_50_ values (4.0 and 4.9 nM against two different viral strains, C-C chemokine receptor type 5 (CCR5)-dependent JRFL and CXC-chemokine receptor 4 (CXCR4)-dependent LAI strains of HIV-1, respectively) and no cytotoxicity to the HeLa host cell line, with a 20-fold improvement in potency compared to **65** (EC_50_ = 86 ± 24 nM in CXCR4-dependent LAI strain of HIV-1) [188]. 

Despite the good antiviral profile, physicochemical properties of these first derivatives were associated with drug formulation and delivery issues. Moderate stability in human liver microsomes (HLM) and low aqueous solubility exerted by **66** may have complicated its preclinical and/or clinical development. In order to improve these features, nitrogen was introduced to the indole ring, generating four azaindole analogues of **66** (**67**–**70**, Table 3) all possessing better PK and pharmaceutical profiles [191]. Interestingly, the indole position onto which the nitrogen was inserted seems to play a crucial role in potency, as exemplified by the 4-aza **67** and the 7-aza **70** isomers, which maintained the antiviral activity compared to **66**, whereas incorporation of the nitrogen atom in a more exposed position of the core was detrimental for HIV-1 inhibitory activity (the 6-aza isomer **69** and the 5-aza analogue **68** were 5- and 100-fold less potent, respectively). Insertion of the nitrogen was instrumental to improving the metabolic stability of isomers **67**–**70** with respect to **66** (half-life (t_1/2_) in HLM: **66** 16.9 min; **67**–**70** from 38.5 to >100 min. The presence of the azaindole ring may also be exploited to convert the compounds into the corresponding salts, facilitating their formulation [191]. The improved basicity displayed by the azaindoles seemed to correlate with their permeability across a Caco-2 monolayer at pH 6.5. Compound 7-azaindole **70** (pKa 2.0) should predominantly exist as a free base, thus possessing high permeability, whereas 4-azaindole **67** (pKa 5.0) could be protonated, leading to reduced permeability. For the same reason, a considerable amount of both 5-azaindole **68** (pKa 6.2) and 6-azaindole **69** (pKa 6.0) would be present as the pyridinium cation at pH 6.5, reducing their ability to penetrate across the Caco-2 membrane. 

Additional development of these azaindoles led to the identification of clinical candidates: 7-azaindole HIV-1 attachment inhibitor BMS-378806 **71** [192] and 6-azaindole derivative BMS-488043 **72** (Table 3) [193]. Low permeability and moderate metabolic stability caused the plasma concentration of **71** to decrease after oral administration in humans, leading to a halt in its development, whereas **72** showed an improved in vivo PK profile in rat, dog, and monkey (**71**: t_1/2_ in HLM 37 min, Caco-2 permeability 51 nm/s; **72**: t_1/2_ in HLM 100 min, Caco-2 permeability 178 nm/s) [191,194]. Compound **72** was able to reduce viremia in HIV-1-infected subjects when administered as a monotherapy, validating the use of HIV-1 inhibitors as a potential treatment of HIV-1 infection in vivo [195]. 

**Figure 22:**
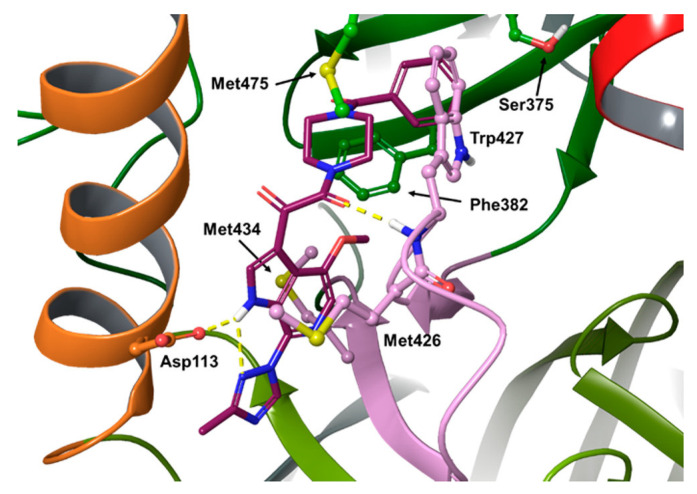
X-ray structure of the co-crystal of the gp120/**73** complex [167]. Reproduced from reference [167]. Copyright 2017 American Chemical Society; https://doi.org/10.2210/pdb5U7O/pdb accessed on 1 May 2023.

Capitalizing on these findings, a subsequent broad optimization campaign on compound **65** led to the identification of temsavir **73** (GSK2616713, Table 4), with enhanced antiviral activity against a spectrum of laboratory strains (Table 4) and good PK [167]. The ability of this compound to bind gp120 was demonstrated by mechanistic studies conducted on an X-ray structure of crystal complex **73**/gp120, showing interactions at the interface between the inner and outer domains under the β20−β21 loop (Figure 22) [196]. Despite a predominance of hydrophobic interactions, crucial interactions involved the indolylglyoxylamide moiety, namely, the H-bonds between the backbone NH of W427 with the oxoamide carbonyl and the azaindole NH and the side chain of D11. The benzamide occupied the gp120 site that was also occupied by W427, pushing both W427 and the β20−β21 loop toward the CD4 binding loop and preventing CD4 binding (Figure 22) [167].

Despite the optimal profile of **73**, the phosphonooxymethyl prodrug fostemsavir **74** (GSK3648934, Table 4) was synthesized to fix emerging problems linked to dissolution and solubility-limited absorption. Compound **74** successfully decreased the viral RNA level in patients compared with those receiving placebo during the first 8 days, with efficacy sustained through 48 weeks, in a phase III clinical trial in patients with limited therapeutic options [197]. Eventually, these results led to the approval of **74** from the Food and Drug Administration in July 2020 for patients with limited treatment options (FDA approves new HIV treatment for patients with limited treatment options (https://www.fda.gov/news-events/press-announcements/fda-approves-new-hiv-treatment-patients-limited-treatment-options (accessed 18 August 2020)).

## 8. Indolylglyoxylamides with Antileishmania Properties

Leishmaniasis is a parasitic disease caused by the Leishmania parasite, which is spread to humans through the bite of infected sand flies. This parasite exists in two different forms: the motile flagellated form (promastigotes), which can be found in the gut of the vector, and the non-flagellated form (amastigotes), which is in the mammalian host and it is responsible for acute disease [198,199]. There are three types of cutaneous leishmaniasis: visceral leishmaniasis (VL), the most lethal if left untreated; cutaneous leishmaniasis (CL); and mucocutaneous leishmaniasis (MCL). According to the World Health Organization (WHO), 12 million people are infected worldwide, and in the next few years a dramatic increase is expected of about 2 million new cases per year, out of which 500,000 will be cases of VL. Today, more than 1 billion people live in areas endemic for leishmaniasis and are at risk of infection. An estimated 30,000 new cases of VL and more than 1 million new cases of CL occur annually (https://www.who.int/news-room/fact-sheets/detail/leishmaniasis). Unfortunately, there are few treatment options for leishmaniasis, and the side effects can be very serious. In view of the foregoing facts, there is still an exigent need to develop novel antileishmanial agents.

**Figure 23:**
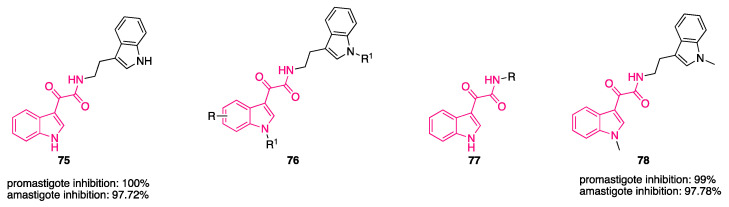
Structures and biological activities of 8,9-dihydrocoscinamide B **75** and its derivatives **76**–**78** as leishmaniasis agents.

Chauhan et al. investigated 8,9-dihydrocoscinamide B (**75**, Figure 23) for its anti-leishmaniasis properties. This molecule is the reduced derivative of coscinamide B, a bis-indole ketoamide isolated from a marine sponge possessing cytoprotective activity against HIV. They also synthesized four analogues maintaining the coscinamide scaffold (**76**, Figure 23) and a small series of indolylglyoxylamide derivatives bearing different amines as substituents of the ketoamide moiety (**77**, Figure 23) [200]. 

The molecules were tested for their inhibitory capacity in both luciferase-promastigote and luciferase-amastigote systems at 10 μg/mL concentration, highlighting the better anti-leishmaniasis activity of coscinamide derivatives compared to the differently substituted indolylglyoxylamides. Particularly, 8,9-dihydrocoscinamide B **75** and the methylated derivative **78** (Figure 23) showed the most potent inhibitory percentage in both systems (100 and 99% in promastigote system; 97.72 and 97.78% in amastigote system) [200].

**Figure 24:**
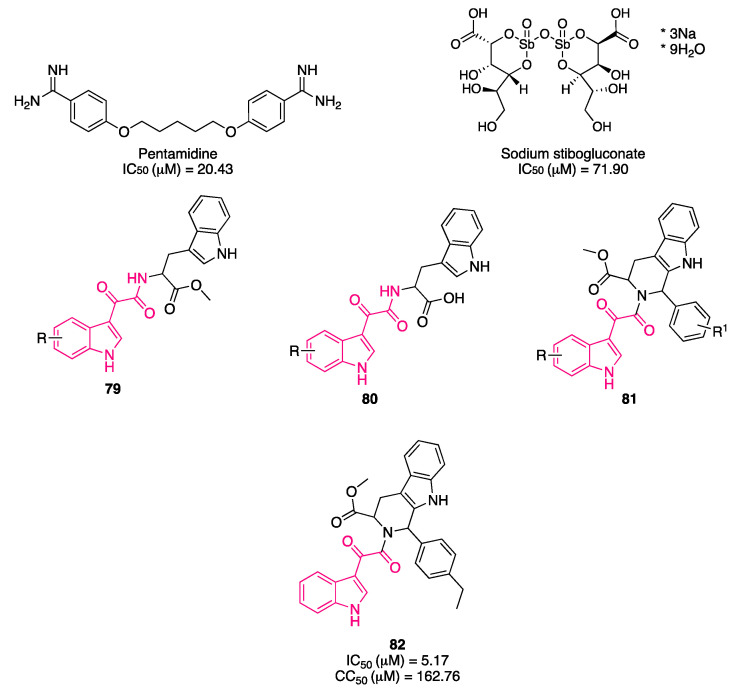
Structures and biological activities of standard drugs pentamidine and sodium stibogluconate, novel tryptophan **79**–**80**, and tetrahydro-β-carboline derivatives **81**–**82** as leishmaniasis agents [204].

In a subsequent study, Chauhan et al. synthesized a series of indolylglyoxylamide derivatives, introducing a carboxylic moiety at the alpha position to the ketoamide nitrogen in the form of either a methyl ester **79** or carboxylic acid **80** (Figure 24). Moreover, prompted by the good anti-leishmaniasis activity shown by some β-carboline derivatives [201,202,203], another series of indolylglyoxylamides **81** bearing various tetrahydro-β-carboline moieties was developed (Figure 24) [204].

Most methyl ester derivatives showed low activity and selectivity when tested for activity in vitro against transgenic *L. donovani* amastigotes. They also showed an unfavorable toxicity profile. Hydrolysis of methyl ester to carboxylic acid led to a complete loss of activity. 

On the contrary, indolylglyoxylamide derivatives **80** displayed good biological activity, with IC_50_ values in the range of 3.79–8.04 µM, thus representing promising candidates for the development of new antileishmanial drugs. The antileishmanial activity was influenced by varying the substitution pattern on the pendant phenyl ring of tetrahydro-β-carbolines, with electron-withdrawing groups more potent than electron-donating ones (**81**). The effect of a withdrawing group on the indole ring was studied by introducing a bromine substituent at position 5, but this led to an increase in toxicity without significantly impacting the activity. Different isomers of tetrahydro-β-carbolines did not affect the activity. Compound **82** (Figure 24), bearing an ethyl group at the *para* position of the pendant phenyl ring, appeared to be the molecule with the best profile, with an IC_50_ value of 5.17 µM, toxicity above 100 μM, and a high selectivity index of 31.48—12- and 5-fold greater than the standard drugs pentamidine and sodium stibogluconate (SSG), respectively [204]. 

## 9. Indolylglyoxylamides with Antiprion Properties

**Figure 25:**
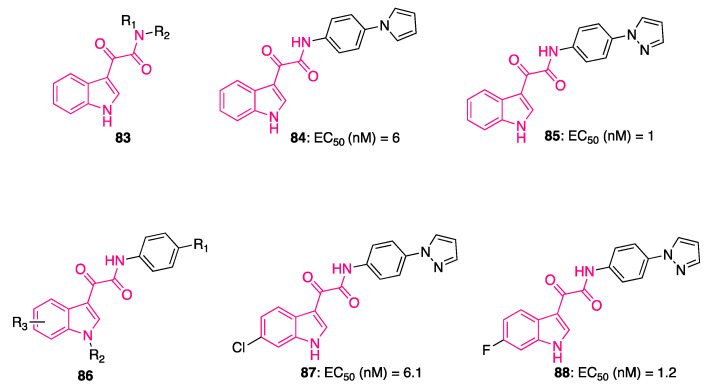
Indolylglyoxylamides **83**–**88** as antiprion agents [207,208].

The indolylglyoxylamide moiety, with its ability to form noncovalent interactions, has found application in the field of antiprion agents, with remarkably potent compounds having been generated. Prion diseases, or transmissible spongiform encephalopathies (TSEs), are a group of progressive neurodegenerative diseases that affect both humans and animals. In TSEs, normal cellular prion protein (PrPC) is converted into an insoluble aggregate conformer PrPSc, in which “Sc” stands for scrapie, the prion disease of sheep and goats, which is thought to be infectious. The death of neuronal cells in TSEs is caused by these aggregates, resulting in vacuolization and the characteristic spongiform degeneration of brain tissue. Despite the high levels of expression in neurons and conservation among mammalian species, the physiological role of PrPC is still generally unclear, even though it appears to be crucially involved in neuroprotection, cell adhesion, and iron metabolism [205,206]. Thompson et al. designed and synthesized a vast series of indol-3-ylglyoxylamides with a general structure **83** (Figure 25). The choice of this structure, obtained from a scrapie-infected mouse brain (SMB) cell line screening assay, was determined by the extensive array of drug candidates bearing this moiety in either the clinical or preclinical stage with different biological activities [2,207].

The compounds’ capability to inhibit PrPSc formation was tested in a prion-infected cell line (SMB) of mesodermal origins, and nanomolar activity was demonstrated only by indol-3-ylglyoxylamide derivatives, featuring a aniline moiety substituted at the *para* position with an aromatic heterocycle bearing at least one hydrogen-bond acceptor (**84** and **85**, EC_50_ 6 nM and 1 nM, respectively, Figure 25) [207]. 

Substitutions at the *C*-4 to *C*-7 positions of the indole ring (**86**, Figure 25) were investigated, which emphasized that only modification at *C*-6 can improve antiprion activity. Specifically, the introduction of strongly electron-withdrawing groups at *C*-6 delivered compounds with an optimal antiprion effect (compounds **87** and **88**, EC_50_ 6.1 nM and 1.2 nM, respectively, Figure 25) [208]. Toxicity profiles were determined in a zebrafish model with more than half of the tested compounds showing no effect on zebrafish survival, including the most potent candidates. Derivatization at R_1_ with methyl or morpholine was not tolerated, yielding a mortality rate of at least 20%. Interestingly, all derivatives substituted at the 6-position displayed enhanced microsomial stability, suggesting that this position is likely a metabolic site for unsubstituted compounds [208]. 

A subsequent series of antiprion agents was synthesized by Thompson et al., first focusing on the *para* substitution of the aniline and then modifying the ketoamide functionality [209]. The results from this investigation corroborated the previous findings that the best phenyl substituent at the *para* position (R_1_) of indol-3-ylglyoxylamide derivatives is a 5-member aromatic ring containing at least two heteroatoms. Additional heteroatoms can be tolerated if at least one is oxygen; further modification of the heterocycle is generally detrimental [209]. 

Detailed modifications were carried out on the glyoxylamide moiety, producing compounds with reduced potency: (1) formation of 3-(aminoacetyl)indoles and indole-3-acetamides by replacing either carbonyl with a methylene group, (2) the introduction of a maleimide bridge, and (3) an increment in the distance between the two carbonyls via the introduction of one or two carbon spacers. After analyzing the two series lacking either the amide carbonyl or the α-keto carbonyl, it was seen that the latter produced a more serious reduction in activity, suggesting its more substantial role in conferring potency to the molecules [209]. 

## 10. Others

### 10.1. A_2B_ Allosteric Modulators

Among adenosine receptors (ARs), the A_2B_ subtype displays lower affinity for adenosine with respect to the other subtypes (A_1_, A_2A_, and A_3_), and it is therefore triggered only at micromolar concentrations of adenosine. Under physiological conditions, extracellular concentration of adenosine is about 100 nM, whereas under stress conditions, it increases to micromolar levels, thus also activating A_2B_AR. A_2B_AR is involved in many physio-pathological conditions, including inflammatory processes, angiogenesis, glucose metabolism, tumor growth, and modulation of the function of cardiac fibroblasts, which are involved in many cardiovascular diseases, like chronic heart failure. For these reasons, A_2B_AR represents an important pharmacological target for a variety of diseases, such as diabetes, tumors, cardiovascular diseases, and pulmonary fibrosis. 

**Figure 26:**
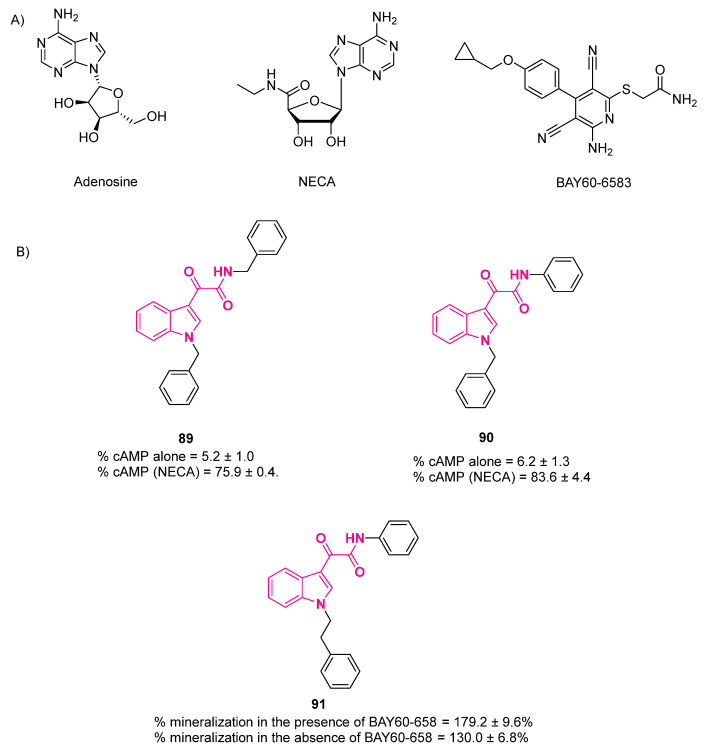
(**A**) Structure of adenosine, NECA, and BAY60-6583; (**B**) indol-3-ylglyoxylamides **89**–**91** as A_2B_AR-positive allosteric modulator agents.

In this context, Taliani and colleagues employed indol-3-ylglyoxylamide to discover novel AR ligands that, surprisingly, acted as A_2B_AR-positive allosteric modulators: compounds **89** and **90** (Figure 26) [210]. Compound **90**, selected as representative for a deeper biological characterization, showed the ability to increase the efficacy but not the potency of A_2B_ agonists, such as adenosine, NECA and BAY60-6583 [211], and the ability to promote MSC differentiation to osteoblasts and bone formation [212].

Subsequently, starting from the basic structures of **89** and **90**, a structurally related series of compounds was developed by the same authors [213]. When evaluated for mineralization activity in the presence of the orthosteric agonist BAY60-6583, the most effective compound was **91** (Figure 26), which showed higher activity with respect to **90**. Of note, compound **91** also significantly enhanced mineralization in the absence of BAY60-6583, thus highlighting its potential use as an anti-osteoporosis agent, as it was able to increase mineralization in experimental conditions close to physio-pathological ones. Finally, functional assays were performed, which confirmed allosteric modulator behavior at A_2B_AR for **91**. Also in this case, **91** enhanced the efficacy of NECA without affecting its potency.

### 10.2. Carbonic Anhydrase Activators

Recently, an involvement of carbonic anhydrase (CA) activation in cognitive and memory disorders was demonstrated [214,215]. In fact, significantly reduced levels of different cerebral isoforms of α-CAs were found in the hippocampus of patients with Alzheimer’s disease. Moreover, following the administration of the activator phenylalanine to experimental animals, a pharmacological improvement in synaptic efficacy, spatial learning, and memory was produced. All of these observations suggest that these enzymes may represent a potential therapeutic target to identify useful agents to treat neurodegenerative diseases. In particular, CA activators (CAAs) act by restoring the active form of the enzyme, working as an alternative or additional site for proton transfer, accelerating the reaction. Exhaustive SARs evidenced a lipophilic aromatic ring substituted with a proton-shuttling moiety by means of a flexible chain as basic structural requirements to develop active CAAs. 

**Figure 27:**
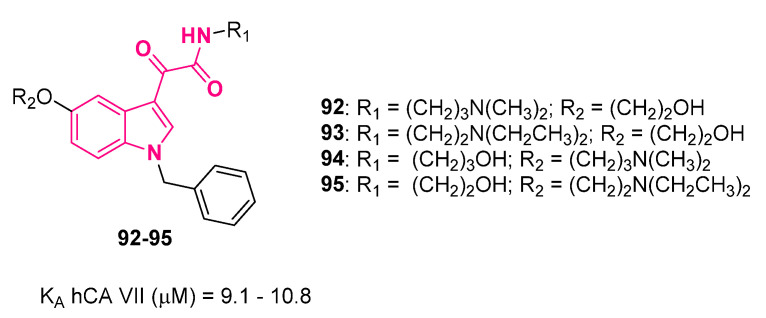
Indol-3-yl-glyoxylamides **92**–**95** as carbonic anhydrase activator agents.

In this connection, a series of indolylglyoxylamides featuring a basic protonable or a polar group at the 3- or 5-position was developed (**92**–**95**, Figure 27) [216]. The choice of this moiety came from the longstanding experience of the research group with the development of indolylglyoxylamides with in vivo and/or in vitro activity [4,35,51,52,57,217] together with the widely recognized role of the alpha-keto amide moiety as a privileged motif in medicinal chemistry [11]. 

Biological assays evidenced that the CA isoform most sensitive to activation by all compounds was the cytosolic isoform VII, also known as the brain-associated isoform, as it is mainly expressed only in the brain [218]. 

The most active compounds were indole-based derivatives featuring at the 3-position a carboxamide or an ethyl ester moiety and the protonable/polar group at the 5-position. These latter compounds increased the release of brain-derived neurotrophic factor (BDNF), acting on the CNS by increasing the growth and differentiation of new neurons [219].

## 11. Conclusions

The aim of this review was to emphasize to medicinal chemists how the indolylglyoxylamide scaffold may serve as a versatile option to develop compounds with different therapeutic applications. Because of its suitability for a vast number of different modifications that enable interaction with specific molecular targets and produce desirable therapeutic effects, indolyglyoxylamide functionality may be considered a unique combination of privileged structures in medicinal chemistry, namely, the indole ring and the α-ketoamide moiety. 

The indole ring features ease of functionalization and the ability both to engage H-bond interactions by means of its nitrogen proton and to establish π-π stacking or cation-π interactions due to its aromaticity. The α-ketoamide moiety may be exploited to modulate the conformation of a lead compound by increasing or decreasing its structural rigidity and to confer additional ability to establish hydrogen bonds in order to improve its potency and/or selectivity to a specific target. As an example, Da Settimo et al. were able to modulate the affinity of indolylglyoxylamide derivatives towards BzR subtypes by varying substituents at the 5-position of the indole and on the ketoamide nitrogen [26,27,28,29,34,35].

Furthermore, the α-ketoamide moiety possesses a unique pharmacokinetic profile showing improved membrane permeability compared to α-ketoacids and enhanced stability toward plasma esterases than α-ketoesters, together with a higher resistance against proteolytic cleavage. As reported by del Rosario García-Lozano et al., the α-ketoamide derivative was not the most potent in terms of antiviral activity against Dengue and Zika viruses but presented a superior predicted pharmacokinetic profile [164].

To increase the potency and/or to enhance the selectivity of a lead compound, as well as to improve its pharmacokinetic properties and/or reduce its toxicity and acquire novel chemical space to secure intellectual property, medicinal chemists may exploit bioisosterism to rationally modify its structure. The new bioisosteric molecule may show structural changes in molecular size, shape, electronic distribution, polarizability, pKa, or dipole that can deeply ameliorate the pharmacological activity of the original compound. In this view, the indolylglyoxylamide moiety may be fruitfully used by medicinal chemists to bioisosterically replace a tricyclic heterocycle to modulate its conformation by decreasing its structural rigidity and, possibly, to confer a new capacity to establish stronger and/or more selective interactions with a target protein. Furthermore, the two α-ketoamide electron-rich oxygen atoms may represent further points of interaction with a target, thus playing a crucial role in enhancing the affinity and selectivity of the compound for a specific protein, especially if the protein is prone to forming hydrogen bonds. In this vein, as an example, Primofiore et al. bioisosterically substituted the β-carboline scaffold with the indolylglyoxylamide one, obtaining highly potent BzR ligands [220]. The indolylglyoxylamide scaffold, having a more flexible structure than a completely planar β-carboline, assumes a planar or pseudoplanar conformation in the interaction with the receptor site, as demonstrated by theoretical calculations showing the indole aromatic ring and the glyoxylamide moiety to lie approximately in the same plane. These results confirm how medicinal chemists may take advantage of the bioisosteric substitution of a tricyclic heteroaromatic scaffold with the more flexible indolylglyoxylamide to develop novel lead compounds with improved pharmacological properties. 

We are aware that in silico methods such as virtual screening (VS) may rapidly provide a set of biologically active compounds in an economically efficient manner and that, very recently, the employment of artificial intelligence methods such as deep and machine learning has added value in small-molecule drug discovery. However, in our opinion, one of the strategies that can aid medicinal chemists in the discovery of new drugs in a shorter amount of time with respect to other strategies is the exploitation of privileged structures and molecular fragments that are able to interact with more than one target. In this overview, we evidenced how the indolylglyoxylamide fragment was exploited in numerous applications to generate biologically active compounds and clinical candidates, primarily as sedative/hypnotics, anxiolytics, antitumorals, antibacterials, antivirals, anti-inflammatories, and antiprions, suggesting its prominent place in drug development. These data unquestionably support how the use of privileged scaffolds in drug design provides access to better success rates by overcoming the intrinsic hurdles connected with time-consuming drug development that rely on structures that are not yet validated.

To conclude, the authors believe that this overview, highlighting indolylglyoxylamide as a unique moiety and emphasizing its peculiar role as valuable option within future drug discovery programs, may aid medicinal chemistry to develop novel agents that are increasingly potent, efficient, and safe.

## Figures and Tables

**Table 1:** Biological activities of compound **12**.

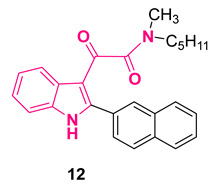		
Activity	Cell Line/Animal Model	Reference
Immunomodulation	Human C20 microglial cells	[58]
Modulation of inflammatory-based retinal neurodegeneration	LPS-induced degeneration in 661W cells	[59]
Neuroprotection	Female mouse model of primary progressive multiple sclerosis	[60]
Maintenance of the correct functionality of microglia in neuroinflammation	Human microglia C20 and HMC3 cells	[61]

**Table 2:** GIIA and group IB (GIB) PLA_2_ inhibition by indol-3-ylglyoxylamides **47**–**50**. Data are taken from Draheim et al. [133].

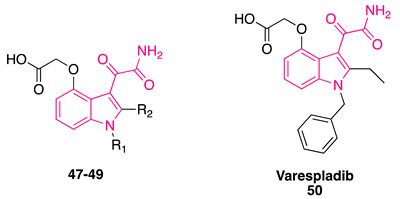					
cpd	R_1_	R_2_	hGIIA PLA_2_ (μM)	hGIB PLA_2_ (μM)	pGIB PLA_2_ (μM)
**47**	2-(C_6_H_5_)C_6_H_4_CH_2_	CH_3_	0.006 ± 0.001	0.364	0.097
**48**	3-(C_6_H_5_)C_6_H_4_CH_2_	CH_3_	0.009 ± 0.001	0.57	0.007
**49**	C_6_H_5_CH_2_	CH_3_	0.011 ± 0.004	0.761	0.015
**50**	--	--	0.009 ± 0.001	0.228	0.048

**Table 3:** Biological activity of HIV-1 attachment inhibitors **65**–**72**. Data are taken from references [167,191].

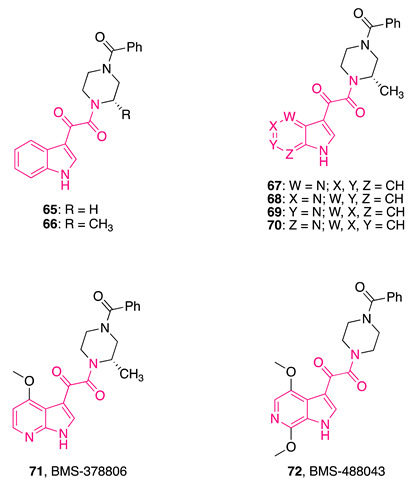		
cpd	EC_50_ (nM)	CC_50_ (μM)
**65**	86 ± 24 (LAI)	145 ± 23
**66**	4.0 (JR-FL) 4.9 (LAI)	200
**67**	1.52 (JR-FL)	>300 (*n* = 2)
**68**	575.9 (JR-FL)	>300 (*n* = 2)
**69**	21.6 (JR-FL)	>300 (*n* = 2)
**70**	1.7 ± 1.6 (JR-FL, *n* = 11)	280
**71**	1.47 ± 0.63 (JR-FL) 2.68 ± 1.64 (LAI)	>300
**72**	0.88 ± 0.46 (JR-FL, *n* = 56) 1.15 (LAI)	>300

**Table 4:** Activity in cellulo of **73** against laboratory strains of HIV-1. Data are taken from Ref. [167].

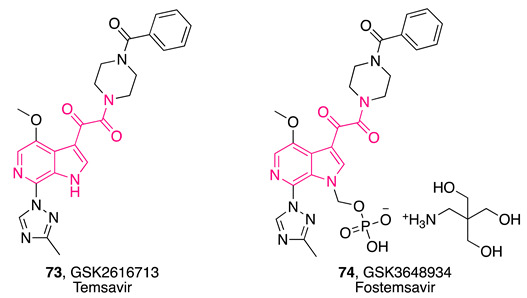		
Co-Receptor Tropism	Virus	EC_50_ (nM)
CCR5	JR-FL	0.4 ± 0.1
	SF-162	0.5 ± 0.2
	Bal	1.7 ± 0.5
CXCR4	LAI	0.7 ± 0.4
	NL4−3	2.2 ± 0.6
	MN	14.8 ± 5.2
	IIIb	16.2 ± 1.7
	RF	>2000

## Data Availability

Not applicable.

## References

[1] Evans B.E., Rittle K.E., Bock M.G., DiPardo R.M., Freidinger R.M., Whitter W.L., Lundell G.F., Veber D.F., Anderson P.S., Chang R.S.L. (1988). Methods for Drug Discovery: Development of Potent, Selective, Orally Effective Cholecystokinin Antagonists. J. Med. Chem..

[2] de Sa Alves F., Barreiro E., Manssour Fraga C. (2009). From Nature to Drug Discovery: The Indole Scaffold as a “Privileged Structure”. Mini Rev. Med. Chem..

[3] Salerno S., Barresi E., Baglini E., Poggetti V., Da Settimo F., Taliani S. (2023). Target-Based Anticancer Indole Derivatives for the Development of Anti-Glioblastoma Agents. Molecules.

[4] Taliani S., Da Settimo F., Martini C., Laneri S., Novellino E., Greco G. (2020). Exploiting the Indole Scaffold to Design Compounds Binding to Different Pharmacological Targets. Molecules.

[5] Shimazaki Y., Yajima T., Takani M., Yamauchi O. (2009). Metal Complexes Involving Indole Rings: Structures and Effects of Metal–Indole Interactions. Coord. Chem. Rev..

[6] Da Settimo F., Lucacchini A., Marini A.M., Martini C., Primofiore G., Senatore G., Taliani S. (1996). Isosteric Replacement of the Indole Nucleus by Benzothiophene and Benzofuran in a Series of Indolylglyoxylylamine Derivatives with Partial Agonist Activity at the Benzodiazepine Receptor. Eur. J. Med. Chem..

[7] Bianucci A.M., Da Settimo A., Da Settimo F., Primofiore G., Martini C., Giannaccini G., Lucacchini A. (1992). Benzodiazepine Receptor Affinity and Interaction of Some N-(Indol-3-Ylglyoxylyl)Amine Derivatives. J. Med. Chem..

[8] Bondensgaard K., Ankersen M., Thøgersen H., Hansen B.S., Wulff B.S., Bywater R.P. (2004). Recognition of Privileged Structures by G-Protein Coupled Receptors. J. Med. Chem..

[9] Rad R., Mracec M., Mracec M., Oprea T. (2007). The Privileged Structures Hypothesis for G Protein-Coupled Receptors—Some Preliminary Results. Rev. Roum. Chim..

[10] Desiraju G.R. (1995). Supramolecular Synthons in Crystal Engineering—A New Organic Synthesis. Angew. Chemie Int. Ed. English.

[11] Robello M., Barresi E., Baglini E., Salerno S., Taliani S., Settimo F. (2021). Da The Alpha Keto Amide Moiety as a Privileged Motif in Medicinal Chemistry: Current Insights and Emerging Opportunities. J. Med. Chem..

[12] Etter M.C. (1990). Encoding and Decoding Hydrogen-Bond Patterns of Organic Compounds. Acc. Chem. Res..

[13] Morin C.M., Jarrin D.C. (2022). Epidemiology of Insomnia: Prevalence, Course, Risk Factors, and Public Health Burden. Sleep Med. Clin..

[14] Mitler M.M. (2000). Nonselective and Selective Benzodiazepine Receptor Agonists—Where Are We Today?. Sleep.

[15] Barbera J., Shaprio C. (2005). Benefit-Risk Assessment of Zaleplon in the Treatment of Insomnia. Drug Saf..

[16] Sanger D.J., Benavides J., Perrault G., Morel E., Cohen C., Joly D., Zivkovic B. (1994). Recent Developments in the Behavioral Pharmacology of Benzodiazepine (ω) Receptors: Evidence for the Functional Significance of Receptor Subtypes. Neurosci. Biobehav. Rev..

[17] Terzano M.G., Rossi M., Palomba V., Smerieri A., Parrino L. (2003). New Drugs for Insomnia: Comparative Tolerability of Zopiclone, Zolpidem and Zaleplon. Drug Saf..

[18] Krystal A.D., Walsh J.K., Laska E., Caron J., Amato D.A., Wessel T.C., Roth T. (2003). Sustained Efficacy of Eszopiclone Over 6 Months of Nightly Treatment: Results of a Randomized, Double-Blind, Placebo-Controlled Study in Adults with Chronic Insomnia. Sleep.

[19] Roth T., Soubrane C., Titeux L., Walsh J.K., on behalf of the Zoladult Study Group (2006). Efficacy and Safety of Zolpidem-MR: A Double-Blind, Placebo-Controlled Study in Adults with Primary Insomnia. Sleep Med..

[20] Farber R.H., Burke P.J. (2008). Post-Bedtime Dosing with Indiplon in Adults and the Elderly: Results from Two Placebo-Controlled, Active Comparator Crossover Studies in Healthy Volunteers. Curr. Med. Res. Opin..

[21] Sieghart W., Sperk G. (2005). Subunit Composition, Distribution and Function of GABA-A Receptor Subtypes. Curr. Top. Med. Chem..

[22] Möhler H., Fritschy J.M., Rudolph U. (2002). A New Benzodiazepine Pharmacology. J. Pharmacol. Exp. Ther..

[23] Whiting P.J. (2003). GABA-A Receptor Subtypes in the Brain: A Paradigm for CNS Drug Discovery?. Drug Discov. Today.

[24] Da Settimo F., Taliani S., Trincavelli M., Montali M., Martini C. (2007). GABAA/Bz Receptor Subtypes as Targets for Selective Drugs. Curr. Med. Chem..

[25] Zhang W., Koehler K.F., Zhang P., Cook J.M. (1995). Development of a Comprehensive Pharmacophore Model for the Benzodiazepine Receptor. Drug Des. Discov..

[26] Da Settimo A., Primofiore G., Da Settimo F., Marini A.M., Novellino E., Greco G., Martini C., Giannaccini G., Lucacchini A. (1996). Synthesis, Structure-Activity Relationships, and Molecular Modeling Studies of N-(Indol-3-Ylglyoxylyl)Benzylamine Derivatives Acting at the Benzodiazepine Receptor. J. Med. Chem..

[27] Primofiore G., Da Settimo F., Taliani S., Marini A.M., Novellino E., Greco G., Lavecchia A., Besnard F., Trincavelli L., Costa B. (2001). Novel N-(Arylalkyl)Indol-3-Ylglyoxylylamides Targeted as Ligands of the Benzodiazepine Receptor: Synthesis, Biological Evaluation, and Molecular Modeling Analysis of the Structure-Activity Relationships. J. Med. Chem..

[28] Primofiore G., Taliani S., Da Settimo F., Marini A.M., La Motta C., Simorini F., Patrizi M.P., Sergianni V., Novellino E., Greco G. (2007). Novel N-Substituted Indol-3-Ylglyoxylamides Probing the LDi and L1/L2 Lipophilic Regions of the Benzodiazepine Receptor Site in Search for Subtype-Selective Ligands. J. Med. Chem..

[29] He X., Huang Q., Ma C., Yu S., McKernan R., Cook J.M. (2000). Pharmacophore/Receptor Models for GABA(A)/BzR Alpha2beta3gamma2, Alpha3beta3gamma2 and Alpha4beta3gamma2 Recombinant Subtypes. Included Volume Analysis and Comparison to Alpha1beta3gamma2, Alpha5beta3gamma2, and Alpha6beta3gamma2 Subtypes. Drug Des. Discov..

[30] Atack J.R. (2003). Anxioselective Compounds Acting at the GABA(A) Receptor Benzodiazepine Binding Site. Curr. Drug Targets CNS Neurol. Disord..

[31] Atack J.R. (2005). The Benzodiazepine Binding Site of GABAA Receptors as a Target for the Development of Novel Anxiolytics. Expert Opin. Investig. Drugs.

[32] Sigel E., Ernst M. (2018). The Benzodiazepine Binding Sites of GABAA Receptors. Trends Pharmacol. Sci..

[33] Skolnick P. (2012). Anxioselective Anxiolytics: On a Quest for the Holy Grail. Trends Pharmacol. Sci..

[34] Primofiore G., Da Settimo F., Marini A.M., Taliani S., La Motta C., Simorini F., Novellino E., Greco G., Cosimelli B., Ehlardo M. (2006). Refinement of the Benzodiazepine Receptor Site Topology by Structure-Activity Relationships of New N-(Heteroarylmethyl)Indol-3- Ylglyoxylamides. J. Med. Chem..

[35] Taliani S., Cosimelli B., Da Settimo F., Marini A.M., La Motta C., Simorini F., Salerno S., Novellino E., Greco G., Cosconati S. (2009). Identification of Anxiolytic/Nonsedative Agents among Indol-3- Ylglyoxylamides Acting as Functionally Selective Agonists at the γ-Aminobutyric Acid-A (GABAA) A2 Benzodiazepine Receptor. J. Med. Chem..

[36] Bourin M., Hascoët M. (2003). The Mouse Light/Dark Box Test. Eur. J. Pharmacol..

[37] Olsen R.W. (2018). GABAA Receptor: Positive and Negative Allosteric Modulators. Neuropharmacology.

[38] Papadopoulos V., Baraldi M., Guilarte T.R., Knudsen T.B., Lacapère J.J., Lindemann P., Norenberg M.D., Nutt D., Weizman A., Zhang M.R. (2006). Translocator Protein (18 KDa): New Nomenclature for the Peripheral-Type Benzodiazepine Receptor Based on Its Structure and Molecular Function. Trends Pharmacol. Sci..

[39] Taliani S., Da Settimo F., Da Pozzo E., Chelli B., Martini C. (2009). Translocator Protein Ligands as Promising Therapeutic Tools for Anxiety Disorders. Curr. Med. Chem..

[40] Le Fur G., Vaucher N., Perrier M.L., Flamier A., Benavides J., Renault C., Dubroeucq M.C., Guérémy C., Uzan A. (1983). Differentiation between Two Ligands for Peripheral Benzodiazepine Binding Sites, [^3^H]R05-4864 and [^3^H]PK 11195, by Thermodynamic Studies. Life Sci..

[41] Romeo E., Auta J., Kozikowski A.P., Ma D., Papadopoulos V., Puia G., Costa E., Guidotti A. (1992). 2-Aryl-3-Indoleacetamides (FGIN-1): A New Class of Potent and Specific Ligands for the Mitochondrial DBI Receptor (MDR). J. Pharmacol. Exp. Ther..

[42] Kozikowski A.P., Ma D., Brewer J., Sun S., Costa E., Romeo E., Guidotti A. (1993). Chemistry, Binding Affinities, and Behavioral Properties of a New Class of “Antineophobic” Mitochondrial DBI Receptor Complex (MDRC) Ligands. J. Med. Chem..

[43] Costa B., Da Pozzo E., Martini C. (2018). Translocator Protein and Steroidogenesis. Biochem. J..

[44] Papadopoulos V., Lecanu L., Brown R.C., Han Z., Yao Z.X. (2006). Peripheral-Type Benzodiazepine Receptor in Neurosteroid Biosynthesis, Neuropathology and Neurological Disorders. Neuroscience.

[45] Nothdurfter C., Rammes G., Baghai T.C., Schüle C., Schumacher M., Papadopoulos V., Rupprecht R. (2012). Translocator Protein (18 KDa) as a Target for Novel Anxiolytics with a Favourable Side-Effect Profile. J. Neuroendocrinol..

[46] Nothdurfter C., Rupprecht R., Rammes G. (2012). Recent Developments in Potential Anxiolytic Agents Targeting GABAA/BzR Complex or the Translocator Protein (18 kDa) (TSPO). Curr. Top. Med. Chem..

[47] Primofiore G., Da Settimo F., Taliani S., Simorini F., Patrizi M.P., Novellino E., Greco G., Abignente E., Costa B., Chelli B. (2004). N,N-Dialkyl-2-Phenylindol-3-Ylglyoxylamides. A New Class of Potent and Selective Ligands at the Peripheral Renzodiazepine Receptor. J. Med. Chem..

[48] Da Settimo F., Simorini F., Taliani S., La Motta C., Marini A.M., Salerno S., Bellandi M., Novellino E., Greco G., Cosimelli B. (2008). Anxiolytic-like Effects of N,N-Dialkyl-2-Phenylindol-3-Ylglyoxylamides by Modulation of Translocator Protein Promoting Neurosteroid Biosynthesis. J. Med. Chem..

[49] Barresi E., Bruno A., Taliani S., Cosconati S., Da Pozzo E., Salerno S., Simorini F., Daniele S., Giacomelli C., Marini A.M. (2015). Deepening the Topology of the Translocator Protein Binding Site by Novel N,N-Dialkyl-2-Arylindol-3-Ylglyoxylamides. J. Med. Chem..

[50] Barresi E., Robello M., Costa B., Da Pozzo E., Baglini E., Salerno S., Da Settimo F., Martini C., Taliani S. (2021). An Update into the Medicinal Chemistry of Translocator Protein (TSPO) Ligands. Eur. J. Med. Chem..

[51] Da Pozzo E., Giacomelli C., Costa B., Cavallini C., Taliani S., Barresi E., Da Settimo F., Martini C. (2016). TSPO PIGA Ligands Promote Neurosteroidogenesis and Human Astrocyte Well-Being. Int. J. Mol. Sci..

[52] Costa B., Da Pozzo E., Chelli B., Simola N., Morelli M., Luisi M., Maccheroni M., Taliani S., Simorini F., Da Settimo F. (2011). Anxiolytic Properties of a 2-Phenylindolglyoxylamide TSPO Ligand: Stimulation of In Vitro Neurosteroid Production Affecting GABAA Receptor Activity. Psychoneuroendocrinology.

[53] Tu L.N., Morohaku K., Manna P.R., Pelton S.H., Butler W.R., Stocco D.M., Selvaraj V. (2014). Peripheral Benzodiazepine Receptor/Translocator Protein Global Knock-out Mice Are Viable with No Effects on Steroid Hormone Biosynthesis. J. Biol. Chem..

[54] Morohaku K., Pelton S.H., Daugherty D.J., Butler W.R., Deng W., Selvaraj V. (2014). Translocator Protein/Peripheral Benzodiazepine Receptor Is Not Required for Steroid Hormone Biosynthesis. Endocrinology.

[55] M Scarf A., M Auman K., Kassiou M. (2012). Is There Any Correlation between Binding and Functional Effects at the Translocator Protein (TSPO) (18 KDa)?. Curr. Mol. Med..

[56] Costa B., Da Pozzo E., Giacomelli C., Barresi E., Taliani S., Da Settimo F., Martini C. (2016). TSPO Ligand Residence Time: A New Parameter to Predict Compound Neurosteroidogenic Efficacy. Sci. Rep..

[57] Bruno A., Barresi E., Simola N., Da Pozzo E., Costa B., Novellino E., Da Settimo F., Martini C., Taliani S., Cosconati S. (2019). Unbinding of Translocator Protein 18 KDa (TSPO) Ligands: From In Vitro Residence Time to In Vivo Efficacy via in Silico Simulations. ACS Chem. Neurosci..

[58] Germelli L., Da Pozzo E., Giacomelli C., Tremolanti C., Marchetti L., Wetzel C.H., Barresi E., Taliani S., Settimo F.D., Martini C. (2021). De Novo Neurosteroidogenesis in Human Microglia: Involvement of the 18 Kda Translocator Protein. Int. J. Mol. Sci..

[59] Corsi F., Baglini E., Barresi E., Salerno S., Cerri C., Martini C., Da Settimo Passetti F., Taliani S., Gargini C., Piano I. (2022). Targeting TSPO Reduces Inflammation and Apoptosis in an In Vitro Photoreceptor-Like Model of Retinal Degeneration. ACS Chem. Neurosci..

[60] Tremolanti C., Cavallini C., Meyer L., Klein C., Da Pozzo E., Costa B., Germelli L., Taliani S., Patte-Mensah C., Mensah-Nyagan A.G. (2022). Translocator Protein Ligand PIGA1138 Reduces Disease Symptoms and Severity in Experimental Autoimmune Encephalomyelitis Model of Primary Progressive Multiple Sclerosis. Mol. Neurobiol..

[61] Angeloni E., Germelli L., Marchetti L., Da Pozzo E., Tremolanti C., Wetzel C.H., Baglini E., Taliani S., Da Settimo F., Martini C. (2023). The Human Microglial Surveillant Phenotype Is Preserved by de Novo Neurosteroidogenesis through the Control of Cholesterol Homeostasis: Crucial Role of 18 KDa Translocator Protein. Biochim. Biophys. Acta Mol. Basis Dis..

[62] Chhikara B.S., Parang K. (2023). Global Cancer Statistics 2022: The Trends Projection Analysis. Chem. Biol. Lett..

[63] Ganesh K., Massagué J. (2021). Targeting Metastatic Cancer. Nat. Med..

[64] Mothersill C., Seymour C.B. (1998). Mechanisms and Implications of Genomic Instability and Other Delayed Effects of Ionizing Radiation Exposure. Mutagenesis.

[65] Charmsaz S., Collins D.M., Perry A.S., Prencipe M. (2019). Novel Strategies for Cancer Treatment: Highlights from the 55th IACR Annual Conference. Cancers.

[66] Shams M., Owczarczak B., Manderscheid-Kern P., Bellnier D.A., Gollnick S.O. (2015). Development of Photodynamic Therapy Regimens That Control Primary Tumor Growth and Inhibit Secondary Disease. Cancer Immunol. Immunother..

[67] Allen C., Her S., Jaffray D.A. (2017). Radiotherapy for Cancer: Present and Future. Adv. Drug Deliv. Rev..

[68] Yahya E.B., Alqadhi A.M. (2021). Recent Trends in Cancer Therapy: A Review on the Current State of Gene Delivery. Life Sci..

[69] Bukowski K., Kciuk M., Kontek R. (2020). Mechanisms of Multidrug Resistance in Cancer Chemotherapy. Int. J. Mol. Sci..

[70] Jordan M.A. (2002). Mechanism of Action of Antitumor Drugs That Interact with Microtubules and Tubulin. Curr. Med. Chem. Anticancer Agents.

[71] Bacher G., Nickel B., Emig P., Vanhoefer U., Seeber S., Shandra A., Klenner T., Beckers T. (2001). D-24851, a Novel Synthetic Microtubule Inhibitor, Exerts Curative Antitumoral Activity In Vivo, Shows Efficacy toward Multidrug-Resistant Tumor Cells, and Lacks Neurotoxicity. Cancer Res..

[72] Windebank A.J. (1999). Chemotherapeutic Neuropathy: Current Opinion in Neurology. Curr. Opin. Neurol..

[73] Rowinsky E.K. (1997). The Development and Clinical Utility of the Taxane Class of Antimicrotubule Chemotherapy Agents. Annu. Rev. Med..

[74] Kuppens I.E.L.M., Witteveen P.O., Schot M., Schuessler V.M., Daehling A., Beijnen J.H., Voest E.E., Schellens J.H.M. (2007). Phase I Dose-Finding and Pharmacokinetic Trial of Orally Administered Indibulin (D-24851) to Patients with Solid Tumors. Invest. New Drugs.

[75] Oostendorp R.L., Witteveen P.O., Schwartz B., Vainchtein L.D., Schot M., Nol A., Rosing H., Beijnen J.H., Voest E.E., Schellens J.H.M. (2010). Dose-Finding and Pharmacokinetic Study of Orally Administered Indibulin (D-24851) to Patients with Advanced Solid Tumors. Invest. New Drugs.

[76] Sorgel F., Kinzig M. (1993). Pharmacokinetics of Gyrase Inhibitors, Part 2: Renal and Hepatic Elimination Pathways and Drug Interactions. Am. J. Med..

[77] Hagen S.E., Domagala J., Gajda C., Lovdahl M., Tait B.D., Wise E., Holler T., Hupe D., Nouhan C., Urumov A. (2001). 4-Hydroxy-5,6-Dihydropyrones as Inhibitors of HIV Protease: The Effect of Heterocyclic Substituents at C-6 on Antiviral Potency and Pharmacokinetic Parameters. J. Med. Chem..

[78] Li W.T., Hwang D.R., Chen C.P., Shen C.W., Huang C.L., Chen T.W., Lin C.H., Chang Y.L., Chang Y.Y., Lo Y.K. (2003). Synthesis and Biological Evaluation of N-Heterocyclic Indolyl Glyoxylamides as Orally Active Anticancer Agents. J. Med. Chem..

[79] Hu C.B., Chen C.P., Yeh T.K., Song J.S., Chang C.Y., Chuu J.J., Tung F.F., Ho P.Y., Chen T.W., Lin C.H. (2011). BPR0C261 Is a Novel Orally Active Antitumor Agent with Antimitotic and Anti-Angiogenic Activities. Cancer Sci..

[80] Leu J.D., Lin S.T., Chen C.T., Chang C.A., Lee Y.J. (2022). BPR0C261, An Analogous of Microtubule Disrupting Agent D-24851 Enhances the Radiosensitivity of Human Non-Small Cell Lung Cancer Cells via P53-Dependent and P53-Independent Pathways. Int. J. Mol. Sci..

[81] Vogelstein B., Lane D., Levine A.J. (2000). Surfing the P53 Network. Nature.

[82] Choy H. (2001). Taxanes in Combined Modality Therapy for Solid Tumors. Crit. Rev. Oncol. Hematol..

[83] Li W.T., Yeh T.K., Song J.S., Yang Y.N., Chen T.W., Lin C.H., Chen C.P., Shen C.C., Hsieh C.C., Lin H.L. (2013). BPR0C305, an Orally Active Microtubule-Disrupting Anticancer Agent. Anticancer. Drugs.

[84] Colley H.E., Muthana M., Danson S.J., Jackson L.V., Brett M.L., Harrison J., Coole S.F., Mason D.P., Jennings L.R., Wong M. (2015). An Orally Bioavailable, Indole-3-Glyoxylamide Based Series of Tubulin Polymerization Inhibitors Showing Tumor Growth Inhibition in a Mouse Xenograft Model of Head and Neck Cancer. J. Med. Chem..

[85] Chen Y.J., Huang W.C., Wei Y.L., Hsu S.C., Yuan P., Lin H.Y., Wistuba I.I., Lee J.J., Yen C.J., Su W.C. (2011). Elevated BCRP/ABCG2 Expression Confers Acquired Resistance to Gefitinib in Wild-Type EGFR-Expressing Cells. PLoS ONE.

[86] Guggilapu S.D., Lalita G., Reddy T.S., Prajapti S.K., Nagarsenkar A., Ramu S., Brahma U.R., Lakshmi U.J., Vegi G.M.N., Bhargava S.K. (2017). Synthesis of C5-Tethered Indolyl-3-Glyoxylamide Derivatives as Tubulin Polymerization Inhibitors. Eur. J. Med. Chem..

[87] Guggilapu S.D., Guntuku L., Reddy T.S., Nagarsenkar A., Sigalapalli D.K., Naidu V.G.M., Bhargava S.K., Bathini N.B. (2017). Synthesis of Thiazole Linked Indolyl-3-Glyoxylamide Derivatives as Tubulin Polymerization Inhibitors. Eur. J. Med. Chem..

[88] Brel V.K., Artyushin O.I., Chuprov-Netochin R.N., Leonov S.V., Semenova M.N., Semenov V.V. (2020). Synthesis and Biological Evaluation of Indolylglyoxylamide Bisphosphonates, Antimitotic Microtubule-Targeting Derivatives of Indibulin with Improved Aqueous Solubility. Bioorg. Med. Chem. Lett..

[89] Tantak M.P., Gupta V., Nikhil K., Arun V., Singh R.P., Jha P.N., Shah K., Kumar D. (2016). Sequential One-Pot Synthesis of Bis(Indolyl)Glyoxylamides: Evaluation of Antibacterial and Anticancer Activities. Bioorg. Med. Chem. Lett..

[90] Tantak M.P., Wang J., Singh R.P., Kumar A., Shah K., Kumar D. (2015). 2-(3′-Indolyl)-N-Arylthiazole-4-Carboxamides: Synthesis and Evaluation of Antibacterial and Anticancer Activities. Bioorg. Med. Chem. Lett..

[91] Venkataramana Reddy P.O., Tantak M.P., Valdez R., Singh R.P., Singh O.M., Sadana R., Kumar D. (2016). Synthesis and Biological Evaluation of Novel Carbazolyl Glyoxamides as Anticancer and Antibacterial Agents. RSC Adv..

[92] Soni J.P., Nikitha Reddy G., Rahman Z., Sharma A., Spandana A., Phanindranath R., Dandekar M.P., Nagesh N., Shankaraiah N. (2023). Synthesis and Cytotoxicity Evaluation of DNA-Interactive β-Carboline Indolyl-3-Glyoxamide Derivatives: Topo-II Inhibition and in Silico Modelling Studies. Bioorg. Chem..

[93] Tokala R., Thatikonda S., Vanteddu U.S., Sana S., Godugu C., Shankaraiah N. (2018). Design and Synthesis of DNA-Interactive β-Carboline–Oxindole Hybrids as Cytotoxic and Apoptosis-Inducing Agents. ChemMedChem.

[94] Jadala C., Sathish M., Reddy T.S., Reddy V.G., Tokala R., Bhargava S.K., Shankaraiah N., Nagesh N., Kamal A. (2019). Synthesis and In Vitro Cytotoxicity Evaluation of β-Carboline-Combretastatin Carboxamides as Apoptosis Inducing Agents: DNA Intercalation and Topoisomerase-II Inhibition. Bioorg. Med. Chem..

[95] M O’Boyle N., J Meegan M. (2011). Designed Multiple Ligands for Cancer Therapy. Curr. Med. Chem..

[96] Villalonga-Planells R., Coll-Mulet L., Martínez-Soler F., Castaño E., Acebes J.J., Giménez-Bonafé P., Gil J., Tortosa A. (2011). Activation of P53 by Nutlin-3a Induces Apoptosis and Cellular Senescence in Human Glioblastoma Multiforme. PLoS ONE.

[97] Shoukrun R., Veenman L., Shandalov Y., Leschiner S., Spanier I., Karry R., Katz Y., Weisinger G., Weizman A., Gavish M. (2008). The 18-KDa Translocator Protein, Formerly Known as the Peripheral-Type Benzodiazepine Receptor, Confers Proapoptotic and Antineoplastic Effects in a Human Colorectal Cancer Cell Line. Pharmacogenet. Genom..

[98] Daniele S., Taliani S., Da Pozzo E., Giacomelli C., Costa B., Trincavelli M.L., Rossi L., La Pietra V., Barresi E., Carotenuto A. (2014). Apoptosis Therapy in Cancer: The First Single-Molecule Co-Activating P53 and the Translocator Protein in Glioblastoma. Sci. Rep..

[99] Daniele S., La Pietra V., Barresi E., Di Maro S., Da Pozzo E., Robello M., La Motta C., Cosconati S., Taliani S., Marinelli L. (2016). Lead Optimization of 2-Phenylindolylglyoxylyldipeptide Murine Double Minute (MDM)2/Translocator Protein (TSPO) Dual Inhibitors for the Treatment of Gliomas. J. Med. Chem..

[100] Juchum M., Günther M., Laufer S.A. (2015). Fighting Cancer Drug Resistance: Opportunities and Challenges for Mutation-Specific EGFR Inhibitors. Drug Resist. Updat..

[101] Daniele S., Barresi E., Zappelli E., Marinelli L., Novellino E., Settimo F.D., Taliani S., Trincavelli M.L., Martini C., Daniele S. (2016). Long Lasting MDM2/Translocator Protein Modulator: A New Strategy for Irreversible Apoptosis of Human Glioblastoma Cells. Oncotarget.

[102] Wang D., Lippard S.J. (2005). Cellular Processing of Platinum Anticancer Drugs. Nat. Rev. Drug Discov..

[103] Oun R., Moussa Y.E., Wheate N.J. (2018). The Side Effects of Platinum-Based Chemotherapy Drugs: A Review for Chemists. Dalt. Trans..

[104] Johnstone T.C., Suntharalingam K., Lippard S.J. (2016). The Next Generation of Platinum Drugs: Targeted Pt(II) Agents, Nanoparticle Delivery, and Pt(IV) Prodrugs. Chem. Rev..

[105] Aquino M.A.S. (1998). Diruthenium and Diosmium Tetracarboxylates: Synthesis, Physical Properties and Applications. Coord. Chem. Rev..

[106] Barresi E., Tolbatov I., Pratesi A., Notarstefano V., Baglini E., Daniele S., Taliani S., Re N., Giorgini E., Martini C. (2020). A Mixed-Valence Diruthenium(Ii,Iii) Complex Endowed with High Stability: From Experimental Evidence to Theoretical Interpretation. Dalt. Trans..

[107] Barresi E., Tolbatov I., Marzo T., Zappelli E., Marrone A., Re N., Pratesi A., Martini C., Taliani S., Da Settimo F. (2021). Two Mixed Valence Diruthenium(II,III) Isomeric Complexes Show Different Anticancer Properties. Dalt. Trans..

[108] Chiaverini L., Baglini E., Mannelli M., Poggetti V., Da Settimo F., Taliani S., Gamberi T., Barresi E., La Mendola D., Marzo T. (2023). A Complex Bearing TSPO PIGA Ligand Coordinated to the [Au(PEt3)]+ Pharmacophore Is Highly Cytotoxic against Ovarian Cancer Cells. BioMetals.

[109] Landini I., Massai L., Cirri D., Gamberi T., Paoli P., Messori L., Mini E., Nobili S. (2020). Structure-Activity Relationships in a Series of Auranofin Analogues Showing Remarkable Antiproliferative Properties. J. Inorg. Biochem..

[110] Nutma E., Ceyzériat K., Amor S., Tsartsalis S., Millet P., Owen D.R., Papadopoulos V., Tournier B.B. (2021). Cellular Sources of TSPO Expression in Healthy and Diseased Brain. Eur. J. Nucl. Med. Mol. Imaging.

[111] Takhi M., Singh G., Murugan C., Thaplyyal N., Maitra S., Bhaskarreddy K.M., Amarnath P.V.S., Mallik A., Harisudan T., Trivedi R.K. (2008). Novel and Potent Oxazolidinone Antibacterials Featuring 3-Indolylglyoxamide Substituents. Bioorg. Med. Chem. Lett..

[112] Clemett D., Markham A. (2000). Linezolid. Drugs.

[113] Shinabarger D.L., Marotti K.R., Murray R.W., Lin A.H., Melchior E.P., Swaney S.M., Dunyak D.S., Demyan W.F., Buysse J.M. (1997). Mechanism of Action of Oxazolidinones: Effects of Linezolid and Eperezolid on Translation Reactions. Antimicrob. Agents Chemother..

[114] Kloss P., Xiong L., Shinabarger D.L., Mankin A.S. (1999). Resistance Mutations in 23 S RRNA Identify the Site of Action of the Protein Synthesis Inhibitor Linezolid in the Ribosomal Peptidyl Transferase Center. J. Mol. Biol..

[115] Johnson A.P., Tysall L., Stockdale M.W., Woodford N., Kaufmann M.E., Warner M., Livermore D.M., Asboth F., Allerberger F.J. (2002). Emerging Linezolid-Resistant Enterococcus Faecalis and Enterococcus Faecium Isolated from Two Austrian Patients in the Same Intensive Care Unit. Eur. J. Clin. Microbiol. Infect. Dis..

[116] Singh P., Verma P., Yadav B., Komath S.S. (2011). Synthesis and Evaluation of Indole-Based New Scaffolds for Antimicrobial Activities—Identification of Promising Candidates. Bioorg. Med. Chem. Lett..

[117] Mielczarek M., Devakaram R.V., Ma C., Yang X., Kandemir H., Purwono B., Black D.S., Griffith R., Lewis P.J., Kumar N. (2014). Synthesis and Biological Activity of Novel Bis-Indole Inhibitors of Bacterial Transcription Initiation Complex Formation. Org. Biomol. Chem..

[118] Li S.A., Cadelis M.M., Sue K., Blanchet M., Vidal N., Brunel J.M., Bourguet-Kondracki M.L., Copp B.R. (2019). 6-Bromoindolglyoxylamido Derivatives as Antimicrobial Agents and Antibiotic Enhancers. Bioorganic Med. Chem..

[119] Pieri C., Borselli D., Di Giorgio C., De Méo M., Bolla J.M., Vidal N., Combes S., Brunel J.M. (2014). New Ianthelliformisamine Derivatives as Antibiotic Enhancers against Resistant Gram-Negative Bacteria. J. Med. Chem..

[120] Cadelis M.M., Pike E.I.W., Kang W., Wu Z., Bourguet-Kondracki M.L., Blanchet M., Vidal N., Brunel J.M., Copp B.R. (2019). Exploration of the Antibiotic Potentiating Activity of Indolglyoxylpolyamines. Eur. J. Med. Chem..

[121] Conti M., Jin S.L. (1999). The Molecular Biology of Cyclic Nucleotide Phosphodiesterases. Prog. Nucleic Acid Res. Mol. Biol..

[122] Torphy T.J., Stadel J.M., Burman M., Cieslinski L.B., McLaughlin M.M., White J.R., Livi G.P. (1992). Coexpression of Human CAMP-Specific Phosphodiesterase Activity and High Affinity Rolipram Binding in Yeast. J. Biol. Chem..

[123] Compton C.H., Gubb J., Nieman R., Edelson J., Amit O., Bakst A., Ayres J.G., Creemers J.P.H.M., Schultze-Werninghaus G., Brambilla C. (2001). Cilomilast, a Selective Phosphodiesterase-4 Inhibitor for Treatment of Patients with Chronic Obstructive Pulmonary Disease: A Randomised, Dose-Ranging Study. Lancet.

[124] Cave A., Arlett P., Lee E. (1999). Inhaled and Nasal Corticosteroids: Factors Affecting the Risks of Systemic Adverse Effects. Pharmacol. Ther..

[125] Marx D., Tassabehji M., Heer S., Hüttenbrink K.B., Szelenyi I. (2002). Modulation of TNF and GM-CSF Release from Dispersed Human Nasal Polyp Cells and Human Whole Blood by Inhibitors of Different PDE Isoenzymes and Glucocorticoids. Pulm. Pharmacol. Ther..

[126] Kuss H., Hoefgen N., Johanssen S., Kronbach T., Rundfeldt C. (2003). In Vivo Efficacy in Airway Disease Models of N-(3,5-Dichloropyrid-4-Yl)-[1-(4-Fluorobenzyl)-5-Hydroxy-Indole-3-Yl]-Glyoxylic Acid Amide (AWD 12-281), a Selective Phosphodiesterase 4 Inhibitor for Inhaled Administration. J. Pharmacol. Exp. Ther..

[127] Burnouf C., Pruniaux M.-P. (2005). Recent Advances in PDE4 Inhibitors as Immunoregulators and Anti- Inflammatory Drugs. Curr. Pharm. Des..

[128] Barnette M.S. (1999). Phosphodiesterase 4 (PDE4) Inhibitors in Asthma and Chronic Obstructive Pulmonary Disease (COPD). Prog. Drug Res..

[129] Phillips J.E. (2020). Inhaled Phosphodiesterase 4 (PDE4) Inhibitors for Inflammatory Respiratory Diseases. Front. Pharmacol..

[130] Dennis E.A., Cao J., Hsu Y.H., Magrioti V., Kokotos G. (2011). Phospholipase A2 Enzymes: Physical Structure, Biological Function, Disease Implication, Chemical Inhibition, and Therapeutic Intervention. Chem. Rev..

[131] Dillard R.D., Bach N.J., Draheim S.E., Berry D.R., Carlson D.G., Chirgadze N.Y., Clawson D.K., Hartley L.W., Johnson L.M., Jones N.D. (1996). Indole Inhibitors of Human Nonpancreatic Secretory Phospholipase A2. 1. Indole-3-Acetamides. J. Med. Chem..

[132] Dillard R.D., Bach N.J., Draheim S.E., Berry D.R., Carlson D.G., Chirgadze N.Y., Clawson D.K., Hartley L.W., Johnson L.M., Jones N.D. (1996). Indole Inhibitors of Human Nonpancreatic Secretory Phospholipase A2. 2. Indole-3-Acetamides with Additional Functionality. J. Med. Chem..

[133] Draheim S.E., Bach N.J., Dillard R.D., Berry D.R., Carlson D.G., Chirgadze N.Y., Clawson D.K., Hartley L.W., Johnson L.M., Jones N.D. (1996). Indole Inhibitors of Human Nonpancreatic Secretory Phospholipase A2. 3. Indole-3-Glyoxamides. J. Med. Chem..

[134] Mihelich E.D., Schevitz R.W. (1999). Structure-Based Design of a New Class of Anti-Inflammatory Drugs: Secretory Phospholipase A2 Inhibitors, SPI. Biochim. Biophys. Acta Mol. Cell Biol. Lipids.

[135] Abraham E., Naum C., Bandi V., Gervich D., Lowry S.F., Wunderink R., Schein R.M., Macias W., Skerjanec S., Dmitrienko A. (2003). Efficacy and Safety of LY315920Na/S-5920, a Selective Inhibitor of 14-KDa Group IIA Secretory Phospholipase A2, in Patients with Suspected Sepsis and Organ Failure. Crit. Care Med..

[136] Snyder D.W., Bach N.J., Dillard R.D., Draheim S.E., Carlson D.G., Fox N., Roehm N.W., Armstrong C.T., Chang C.H., Hartley L.W. (1999). Pharmacology of LY315920/S-5920, [[3-(Aminooxoacetyl)-2-Ethyl-1-(Phenylmethyl)-1H-Indol-4-Yl]Oxy]Acetate, a Potent and Selective Secretory Phospholipase A2 Inhibitor: A New Class of Anti-Inflammatory Drugs, SPI. J. Pharmacol. Exp. Ther..

[137] Rosenson R.S., Fraser H., Trias J., Hislop C. (2010). Varespladib Methyl in Cardiovascular Disease. Expert Opin. Investig. Drugs.

[138] Bradley J.D., Dmitrienko A.A., Kivitz A.J., Gluck O.S., Weaver A.L., Wiesenhutter C., Myers S.L., Sides G.D. (2005). A Randomized, Double-Blinded, Placebo-Controlled Clinical Trial of LY333013, a Selective Inhibitor of Group II Secretory Phospholipase A 2, in the Treatment of Rheumatoid Arthritis. J. Rheumatol..

[139] Bowton D.L., Dmitrienko A.A., Israel E., Zeiher B.G., Sides G.D. (2005). Impact of a Soluble Phospholipase A2 Inhibitor on Inhaled Allergen Challenge in Subjects with Asthma. J. Asthma.

[140] Rosenson R.S., Hislop C., McConnell D., Elliott M., Stasiv Y., Wang N., Waters D.D. (2009). Effects of 1-H-Indole-3-Glyoxamide (A-002) on Concentration of Secretory Phospholipase A2 (PLASMA Study): A Phase II Double-Blind, Randomised, Placebo-Controlled Trial. Lancet.

[141] Nicholls S.J., Kastelein J.J.P., Schwartz G.G., Bash D., Rosenson R.S., Cavender M.A., Brennan D.M., Koenig W., Jukema J.W., Nambi V. (2014). Varespladib and Cardiovascular Events in Patients With an Acute Coronary Syndrome: The VISTA-16 Randomized Clinical Trial. JAMA.

[142] Schett G., Zwerina J., Firestein G. (2008). The P38 Mitogen-Activated Protein Kinase (MAPK) Pathway in Rheumatoid Arthritis. Ann. Rheum. Dis..

[143] Westra J., C Limburg P. (2006). P38 Mitogen-Activated Protein Kinase (MAPK) in Rheumatoid Arthritis. Mini-Reviews Med. Chem..

[144] Schett G., Stach C., Zwerina J., Voll R., Manger B. (2008). How Antirheumatic Drugs Protect Joints from Damage in Rheumatoid Arthritis. Arthritis Rheum..

[145] Korb A., Tohidast-Akrad M., Cetin E., Axmann R., Smolen J., Schett G. (2006). Differential Tissue Expression and Activation of P38 MAPK α, β, γ, and δ Isoforms in Rheumatoid Arthritis. Arthritis Rheum..

[146] Schett G., Tohidast-Akrad M., Smolen J.S., Schmid B.J., Steiner C.W., Bitzan P., Zenz P., Redlich K., Xu Q., Steiner G. (2000). Activation, Differential Localization, and Regulation of the Stress-Activated Protein Kinases, Extracellular Signal-Regulated Kinase, c-Jun N-Terminal Kinase, and P38 Mitogen-Activated Protein Kinase, in Synovial Tissue and Cells in Rheumatoid Arthritis. Arthritis Rheum..

[147] Pargellis C., Tong L., Churchill L., Cirillo P.F., Gilmore T., Graham A.G., Grob P.M., Hickey E.R., Moss N., Pav S. (2002). Inhibition of P38 MAP Kinase by Utilizing a Novel Allosteric Binding Site. Nat. Struct. Biol..

[148] Mavunkel B.J., Chakravarty S., Perumattam J.J., Dugar S., Lu Q., Liang X. (2000). Indole-Type Derivatives as Inhibitors of P38 Kinase.

[149] Snoonian J.R., Oliver-Shaffer P.-A. (2004). Processes for the Preparation Of N-Heteroaryl-N-Aryl-Amines by Reacting an N-Aryl Carbamic Acid Ester with a Halo-Heteroaryl And Analogous Processes.

[150] Hill R.J., Dabbagh K., Phippard D., Li C., Suttmann R.T., Welch M., Papp E., Song K.W., Chang K.C., Leaffer D. (2008). Pamapimod, a Novel P38 Mitogen-Activated Protein Kinase Inhibitor: Preclinical Analysis of Efficacy and Selectivity. J. Pharmacol. Exp. Ther..

[151] Kuma Y., Sabio G., Bain J., Shpiro N., Márquez R., Cuenda A. (2005). BIRB796 Inhibits All P38 MAPK Isoforms In Vitro and In Vivo. J. Biol. Chem..

[152] Goldstein D.M., Kuglstatter A., Lou Y., Soth M.J. (2010). Selective P38α Inhibitors Clinically Evaluated for the Treatment of Chronic Inflammatory Disorders. J. Med. Chem..

[153] Nikas S.N., Drosos A.A. (2004). SCIO-469 Scios Inc. Curr. Opin. Investig. Drugs.

[154] Ji R.R. (2004). Peripheral and Central Mechanisms of Inflammatory Pain, with Emphasis on MAP Kinases. Curr. Drug Targets. Inflamm. Allergy.

[155] Schindler J.F., Monahan J.B., Smith W.G. (2007). P38 Pathway Kinases as Anti-Inflammatory Drug Targets. J. Dent. Res..

[156] Genovese M.C., Cohen S.B., Wofsy D., Weinblatt M.E., Firestein G.S., Brahn E., Strand V., Baker D.G., Tong S.E. (2011). A 24-Week, Randomized, Double-Blind, Placebo-Controlled, Parallel Group Study of the Efficacy of Oral SCIO-469, a P38 Mitogen-Activated Protein Kinase Inhibitor, in Patients with Active Rheumatoid Arthritis. J. Rheumatol..

[157] Rescher S.L.U., Brunotte L., Faist A., Schloer S.M. (2023). P38-Inhibitors for the Treatment of Coronavirus Infections and/or COVID-19 Cytokine Storm.

[158] Leitmeyer K.C., Vaughn D.W., Watts D.M., Salas R., Villalobos I., de Chacon, Ramos C., Rico-Hesse R. (1999). Dengue Virus Structural Differences That Correlate with Pathogenesis. J. Virol..

[159] Wu H., Bock S., Snitko M., Berger T., Weidner T., Holloway S., Kanitz M., Diederich W.E., Steuber H., Walter C. (2015). Novel Dengue Virus NS2B/NS3 Protease Inhibitors. Antimicrob. Agents Chemother..

[160] Li Z., Zhang J., Li H. (2017). Flavivirus NS2B/NS3 Protease: Structure, Function, and Inhibition. Viral Proteases and Their Inhibitors.

[161] Steuer C., Gege C., Fischl W., Heinonen K.H., Bartenschlager R., Klein C.D. (2011). Synthesis and Biological Evaluation of α-Ketoamides as Inhibitors of the Dengue Virus Protease with Antiviral Activity in Cell-Culture. Bioorg. Med. Chem..

[162] Nie S., Yao Y., Wu F., Wu X., Zhao J., Hua Y., Wu J., Huo T., Lin Y.L., Kneubehl A.R. (2021). Synthesis, Structure-Activity Relationships, and Antiviral Activity of Allosteric Inhibitors of Flavivirus NS2B-NS3 Protease. J. Med. Chem..

[163] Mehand M.S., Al-Shorbaji F., Millett P., Murgue B. (2018). The WHO R&D Blueprint: 2018 Review of Emerging Infectious Diseases Requiring Urgent Research and Development Efforts. Antiviral Res..

[164] del Rosario García-Lozano M., Dragoni F., Gallego P., Mazzotta S., López-Gómez A., Boccuto A., Martínez-Cortés C., Rodríguez-Martínez A., Pérez-Sánchez H., Manuel Vega-Pérez J. (2023). Piperazine-Derived Small Molecules as Potential Flaviviridae NS3 Protease Inhibitors. In Vitro Antiviral Activity Evaluation against Zika and Dengue Viruses. Bioorg. Chem..

[165] Kling A., Jantos K., Mack H., Hornberger W., Drescher K., Nimmrich V., Relo A., Wicke K., Hutchins C.W., Lao Y. (2017). Discovery of Novel and Highly Selective Inhibitors of Calpain for the Treatment of Alzheimer’s Disease: 2-(3-Phenyl-1H-Pyrazol-1-Yl)-Nicotinamides. J. Med. Chem..

[166] Dorababu A. (2020). Indole—A Promising Pharmacophore in Recent Antiviral Drug Discovery. RSC Med. Chem..

[167] Meanwell N.A., Krystal M.R., Nowicka-Sans B., Langley D.R., Conlon D.A., Eastgate M.D., Grasela D.M., Timmins P., Wang T., Kadow J.F. (2018). Inhibitors of HIV-1 Attachment: The Discovery and Development of Temsavir and Its Prodrug Fostemsavir. J. Med. Chem..

[168] Ruwizhi N., Aderibigbe B.A. (2020). Cinnamic Acid Derivatives and Their Biological Efficacy. Int. J. Mol. Sci..

[169] Abd El-Raouf O.M., El-Sayed E.S.M., Manie M.F. (2015). Cinnamic Acid and Cinnamaldehyde Ameliorate Cisplatin-Induced Splenotoxicity in Rats. J. Biochem. Mol. Toxicol..

[170] Wang R., Yang W., Fan Y., Dehaen W., Li Y., Li H., Wang W., Zheng Q., Huai Q. (2019). Design and Synthesis of the Novel Oleanolic Acid-Cinnamic Acid Ester Derivatives and Glycyrrhetinic Acid-Cinnamic Acid Ester Derivatives with Cytotoxic Properties. Bioorg. Chem..

[171] Amano R., Yamashita A., Kasai H., Hori T., Miyasato S., Saito S., Yokoe H., Takahashi K., Tanaka T., Otoguro T. (2017). Cinnamic Acid Derivatives Inhibit Hepatitis C Virus Replication via the Induction of Oxidative Stress. Antivir. Res..

[172] Chen Y., Li Z., Pan P., Lao Z., Xu J., Li Z., Zhan S., Liu X., Wu Y., Wang W. (2021). Cinnamic Acid Inhibits Zika Virus by Inhibiting RdRp Activity. Antiviral Res..

[173] Nitsche C., Behnam M.A.M., Steuer C., Klein C.D. (2012). Retro Peptide-Hybrids as Selective Inhibitors of the Dengue Virus NS2B-NS3 Protease. Antivir. Res..

[174] Nitsche C., Steuer C., Klein C.D. (2011). Arylcyanoacrylamides as Inhibitors of the Dengue and West Nile Virus Proteases. Bioorg. Med. Chem..

[175] Deng J., Li N., Liu H., Zuo Z., Liew O.W., Xu W., Chen G., Tong X., Tang W., Zhu J. (2012). Discovery of Novel Small Molecule Inhibitors of Dengue Viral NS2B-NS3 Protease Using Virtual Screening and Scaffold Hopping. J. Med. Chem..

[176] Guo S., Zhen Y., Zhu Z., Zhou G., Zheng X. (2019). Cinnamic Acid Rescues Behavioral Deficits in a Mouse Model of Traumatic Brain Injury by Targeting MiR-455-3p/HDAC2. Life Sci..

[177] Faucher A.M., Bailey M.D., Beaulieu P.L., Brochu C., Duceppe J.S., Ferland J.M., Ghiro E., Gorys V., Halmos T., Kawai S.H. (2004). Synthesis of BILN 2061, an HCV NS3 Protease Inhibitor with Proven Antiviral Effect in Humans. Org. Lett..

[178] Li F., Lee E.M., Sun X., Wang D., Tang H., Zhou G.C. (2020). Design, Synthesis and Discovery of Andrographolide Derivatives against Zika Virus Infection. Eur. J. Med. Chem..

[179] Barbosa-Lima G., Moraes A.M., da Araújo A.S., da Silva E.T., de Freitas C.S., Vieira Y.R., Marttorelli A., Neto J.C., Bozza P.T., de Souza M.V.N. (2017). 2,8-Bis(Trifluoromethyl)Quinoline Analogs Show Improved Anti-Zika Virus Activity, Compared to Mefloquine. Eur. J. Med. Chem..

[180] Balasubramanian A., Teramoto T., Kulkarni A.A., Bhattacharjee A.K., Padmanabhan R. (2017). Antiviral Activities of Selected Antimalarials against Dengue Virus Type 2 and Zika Virus. Antiviral Res..

[181] de la Guardia C., Stephens D.E., Dang H.T., Quijada M., Larionov O.V., Lleonart R. (2018). Antiviral Activity of Novel Quinoline Derivatives against Dengue Virus Serotype 2. Molecules.

[182] Kaptein S.J.F., Vincetti P., Crespan E., Rivera J.I.A., Costantino G., Maga G., Neyts J., Radi M. (2018). Identification of Broad-Spectrum Dengue/Zika Virus Replication Inhibitors by Functionalization of Quinoline and 2,6-Diaminopurine Scaffolds. ChemMedChem.

[183] Beesetti H., Tyagi P., Medapi B., Krishna V.S., Sriram D., Khanna N., Swaminathan S. (2018). A Quinoline Compound Inhibits the Replication of Dengue Virus Serotypes 1-4 in Vero Cells. Antivir. Ther..

[184] Zhao J., Zhang Y., Wang M., Liu Q., Lei X., Wu M., Guo S., Yi D., Li Q., Ma L. (2021). Quinoline and Quinazoline Derivatives Inhibit Viral RNA Synthesis by SARS-CoV-2 RdRp. ACS Infect. Dis..

[185] Vella S., Palmisano L. (2005). The Global Status of Resistance to Antiretroviral Drugs. Clin. Infect. Dis..

[186] Qian K., Morris-Natschke S.L., Lee K.H. (2009). HIV Entry Inhibitors and Their Potential in HIV Therapy. Med. Res. Rev..

[187] Wang T., Zhang Z., Wallace O.B., Deshpande M., Fang H., Yang Z., Zadjura L.M., Tweedie D.L., Huang S., Zhao F. (2003). Discovery of 4-Benzoyl-1-[(4-Methoxy-1H-Pyrrolo [2,3-b]Pyridin-3-Yl)Oxoacetyl]-2-(R) -Methylpiperazine (BMS-378806): A Novel HIV-1 Attachment Inhibitor That Interferes with CD4-Gp120 Interactions. J. Med. Chem..

[188] Meanwell N.A., Wallace O.B., Fang H., Wang H., Deshpande M., Wang T., Yin Z., Zhang Z., Pearce B.C., James J. (2009). Inhibitors of HIV-1 Attachment. Part 2: An Initial Survey of Indole Substitution Patterns. Bioorg. Med. Chem. Lett..

[189] Meanwell N.A., Wallace O.B., Wang H., Deshpande M., Pearce B.C., Trehan A., Yeung K.S., Qiu Z., Wright J.J.K., Robinson B.A. (2009). Inhibitors of HIV-1 Attachment. Part 3: A Preliminary Survey of the Effect of Structural Variation of the Benzamide Moiety on Antiviral Activity. Bioorg. Med. Chem. Lett..

[190] Wang T., Kadow J.F., Zhang Z., Yin Z., Gao Q., Wu D., Parker D.D.G., Yang Z., Zadjura L., Robinson B.A. (2009). Inhibitors of HIV-1 Attachment. Part 4: A Study of the Effect of Piperazine Substitution Patterns on Antiviral Potency in the Context of Indole-Based Derivatives. Bioorg. Med. Chem. Lett..

[191] Wang T., Yin Z., Zhang Z., Bender J.A., Yang Z., Johnson G., Yang Z., Zadjura L.M., D’Arienzo C.J., Parker D.D.G. (2009). Inhibitors of Human Immunodeficiency Virus Type 1 (HIV-1) Attachment. 5. An Evolution from Indole to Azaindoles Leading to the Discovery of 1-(4-Benzoylpiperazin-1-Yl)-2-(4,7-Dimethoxy-1H-Pyrrolo[2,3-c]-Pyridin-3-Yl) Ethane-1,2-Dione (BMS-488043), a Drug. J. Med. Chem..

[192] Lin P.F., Blair W., Wang T., Spicer T., Guo Q., Zhou N., Gong Y.F., Wang H.G.H., Rose R., Yamanaka G. (2003). A Small Molecule HIV-1 Inhibitor That Targets the HIV-1 Envelope and Inhibits CD4 Receptor Binding. Proc. Natl. Acad. Sci. USA.

[193] Ho H.-T., Fan L., Nowicka-Sans B., McAuliffe B., Li C.-B., Yamanaka G., Zhou N., Fang H., Dicker I., Dalterio R. (2006). Envelope Conformational Changes Induced by Human Immunodeficiency Virus Type 1 Attachment Inhibitors Prevent CD4 Binding and Downstream Entry Events. J. Virol..

[194] Yang Z., Zadjura L., D’Arienzo C., Marino A., Santone K., Klunk L., Greene D., Lin P.F., Colonno R., Wang T. (2005). Preclinical Pharmacokinetics of a Novel HIV-1 Attachment Inhibitor BMS-378806 and Prediction of Its Human Pharmacokinetics. Biopharm. Drug Dispos..

[195] Hanna G.J., Lalezari J., Hellinger J.A., Wohl D.A., Nettles R., Persson A., Krystal M., Lin P., Colonno R., Grasela D.M. (2011). Antiviral Activity, Pharmacokinetics, and Safety of BMS-488043, a Novel Oral Small-Molecule HIV-1 Attachment Inhibitor, in HIV-1-Infected Subjects. Antimicrob. Agents Chemother..

[196] Pancera M., Lai Y.T., Bylund T., Druz A., Narpala S., O’Dell S., Schön A., Bailer R.T., Chuang G.Y., Geng H. (2017). Crystal Structures of Trimeric HIV Env with Entry Inhibitors BMS-378806 and BMS-626529. Nat. Chem. Biol..

[197] Kozal M., Aberg J., Pialoux G., Cahn P., Thompson M., Molina J.-M., Grinsztejn B., Diaz R., Castagna A., Kumar P. (2020). Fostemsavir in Adults with Multidrug-Resistant HIV-1 Infection. N. Engl. J. Med..

[198] Akopyants N.S., Kimblin N., Secundino N., Patrick R., Peters N., Lawyer P., Dobson D.E., Beverley S.M., Sacks D.L. (2009). Demonstration of Genetic Exchange during Cyclical Development of Leishmania in the Sand Fly Vector. Science.

[199] Rogers M., Kropf P., Choi B.S., Dillon R., Podinovskaia M., Bates P., Müller I. (2009). Proteophosophoglycans Regurgitated by Leishmania-Infected Sand Flies Target the L-Arginine Metabolism of Host Macrophages to Promote Parasite Survival. PLoS Pathog..

[200] Gupta L., Talwar A., Nishi, Palne S., Gupta S., Chauhan P.M.S. (2007). Synthesis of Marine Alkaloid: 8,9-Dihydrocoscinamide B and Its Analogues as Novel Class of Antileishmanial Agents. Bioorg. Med. Chem. Lett..

[201] Kumar A., Katiyar S.B., Gupta S., Chauhan P.M.S. (2006). Syntheses of New Substituted Triazino Tetrahydroisoquinolines and β-Carbolines as Novel Antileishmanial Agents. Eur. J. Med. Chem..

[202] Costa E.V., Pinheiro M.L.B., Xavier C.M., Silva J.R.A., Amaral A.C.F., Souza A.D.L., Barison A., Campos F.R., Ferreira A.G., Machado G.M.C. (2006). A Pyrimidine-β-Carboline and Other Alkaloids from Annona Foetida with Antileishmanial Activity. J. Nat. Prod..

[203] Kumar R., Khan S., Verma A., Srivastava S., Viswakarma P., Gupta S., Meena S., Singh N., Sarkar J., Chauhan P.M.S. (2010). Synthesis of 2-(Pyrimidin-2-Yl)-1-Phenyl-2,3,4,9-Tetrahydro-1H-β-Carbolines as Antileishmanial Agents. Eur. J. Med. Chem..

[204] Chauhan S.S., Gupta L., Mittal M., Vishwakarma P., Gupta S., Chauhan P.M.S. (2010). Synthesis and Biological Evaluation of Indolyl Glyoxylamides as a New Class of Antileishmanial Agents. Bioorganic Med. Chem. Lett..

[205] Singh A., Mohan M.L., Isaac A.O., Luo X., Petrak J., Vyoral D., Singh N. (2009). Prion Protein Modulates Cellular Iron Uptake: A Novel Function with Implications for Prion Disease Pathogenesis. PLoS ONE.

[206] Hu W., Kieseier B., Frohman E., Eagar T.N., Rosenberg R.N., Hartung H.P., Stüve O. (2008). Prion Proteins: Physiological Functions and Role in Neurological Disorders. J. Neurol. Sci..

[207] Thompson M.J., Borsenberger V., Louth J.C., Judd K.E., Chen B. (2009). Design, Synthesis, and Structure—Activity Relationship of Indole-3-Glyoxylamide Libraries Possessing Highly Potent Activity in a Cell Line Model of Prion Disease. J. Med. Chem..

[208] Thompson M.J., Louth J.C., Ferrara S., Jackson M.P., Sorrell F.J., Cochrane E.J., Gever J., Baxendale S., Silber B.M., Roehl H.H. (2011). Discovery of 6-Substituted Indole-3-Glyoxylamides as Lead Antiprion Agents with Enhanced Cell Line Activity, Improved Microsomal Stability and Low Toxicity. Eur. J. Med. Chem..

[209] Thompson M.J., Louth J.C., Ferrara S., Sorrell F.J., Irving B.J., Cochrane E.J., Meijer A.J.H.M., Chen B. (2011). Structure–Activity Relationship Refinement and Further Assessment of Indole-3-Glyoxylamides as a Lead Series against Prion Disease. ChemMedChem.

[210] Taliani S., Trincavelli M.L., Cosimelli B., Laneri S., Severi E., Barresi E., Pugliesi I., Daniele S., Giacomelli C., Greco G. (2013). Modulation of A2B Adenosine Receptor by 1-Benzyl-3-Ketoindole Derivatives. Eur. J. Med. Chem..

[211] Trincavelli M.L., Giacomelli C., Daniele S., Taliani S., Cosimelli B., Laneri S., Severi E., Barresi E., Pugliesi I., Greco G. (2014). Allosteric Modulators of Human A2B Adenosine Receptor. Biochim. Biophys. Acta Gen. Subj..

[212] Trincavelli M.L., Daniele S., Giacomelli C., Taliani S., Da Settimo F., Cosimelli B., Greco G., Novellino E., Martini C. (2014). Osteoblast Differentiation and Survival: A Role for A2B Adenosine Receptor Allosteric Modulators. Biochim. Biophys. Acta Mol. Cell Res..

[213] Barresi E., Giacomelli C., Marchetti L., Baglini E., Salerno S., Greco G., Da Settimo F., Martini C., Trincavelli M.L., Taliani S. (2021). Novel Positive Allosteric Modulators of A2B Adenosine Receptor Acting as Bone Mineralisation Promoters. J. Enzyme Inhib. Med. Chem..

[214] Supuran C.T. (2008). Carbonic Anhydrases: Novel Therapeutic Applications for Inhibitors and Activators. Nat. Rev. Drug Discov..

[215] Supuran C.T. (2018). Carbonic Anhydrase Activators. Future Med. Chem..

[216] Barresi E., Ravichandran R., Germelli L., Angeli A., Baglini E., Salerno S., Marini A.M., Costa B., Da Pozzo E., Martini C. (2021). Carbonic Anhydrase Activation Profile of Indole-Based Derivatives. J. Enzyme Inhib. Med. Chem..

[217] Santoro A., Mattace Raso G., Taliani S., Da Pozzo E., Simorini F., Costa B., Martini C., Laneri S., Sacchi A., Cosimelli B. (2016). TSPO-Ligands Prevent Oxidative Damage and Inflammatory Response in C6 Glioma Cells by Neurosteroid Synthesis. Eur. J. Pharm. Sci..

[218] Ruusuvuori E., Huebner A.K., Kirilkin I., Yukin A.Y., Blaesse P., Helmy M., Jung Kang H., El Muayed M., Christopher Hennings J., Voipio J. (2013). Neuronal Carbonic Anhydrase VII Provides GABAergic Excitatory Drive to Exacerbate Febrile Seizures. EMBO J..

[219] Matthews T.A., Abel A., Demme C., Sherman T., Pan P.W., Halterman M.W., Parkkila S., Nehrke K. (2014). Expression of the CHOP-Inducible Carbonic Anhydrase CAVI-b Is Required for BDNF-Mediated Protection from Hypoxia. Brain Res..

[220] Primofiore G., Marini A.M., Settimo F.D., Martini C., Bardellini A., Giannaccini G., Lucacchini A. (1989). Specific Inhibition of Benzodiazepine Receptor Binding by Some N-(Indo1-3-Ylglyoxylyl)Amino Acid Derivatives: Stereoselective Interactions. J. Med. Chem..

